# Health and Socioeconomic Factors in School Readiness and Achievement Among Children Born Very Preterm

**DOI:** 10.1001/jamanetworkopen.2026.23068

**Published:** 2026-07-14

**Authors:** Sadia Haider, Athanasios Tsanas, G. David Batty, Rebecca M. Reynolds, Melvyn Roffe, Heather C. Whalley, Riccardo E. Marioni, Hilary Richardson, Cheryl Battersby, James P. Boardman

**Affiliations:** 1 Section of Neonatal Medicine, School of Public Health, Faculty of Medicine, Imperial College London, London, United Kingdom; 2 Usher Institute, Edinburgh Medical School, University of Edinburgh, Edinburgh, United Kingdom; 3 School of Mathematics, University of Edinburgh, United Kingdom; 4 Department of Epidemiology and Public Health, University College London, London, United Kingdom; 5 Centre for Reproductive Health, Institute for Regeneration and Repair, University of Edinburgh, Edinburgh, United Kingdom; 6 Centre for Cardiovascular Science, University of Edinburgh, Edinburgh, United Kingdom; 7 Haberdashers’ Monmouth School, Monmouth, United Kingdom; 8 Centre for Clinical Brain Sciences, University of Edinburgh, Edinburgh, United Kingdom; 9 Centre for Genomic and Experimental Medicine, Institute of Genetics and Cancer, University of Edinburgh, Edinburgh, United Kingdom; 10 School of Philosophy, Psychology, and Language Sciences, University of Edinburgh, Edinburgh, United Kingdom

## Abstract

**Importance:**

Preterm birth is a leading cause of atypical brain development and cognitive impairment; however, there are sparse data on its association with statutory educational assessments.

**Objective:**

To evaluate school readiness at age 5 years and educational attainment at age 6 to 7 years in children born very preterm and to identify the early-life, neonatal, and socioeconomic factors associated with attainment.

**Design, Setting, and Participants:**

This retrospective cohort study included all infants born before gestational age (GA) 32 weeks in England who received care in a neonatal unit and survived to discharge. A linkage between the National Neonatal Research Database and the National Pupil Database was created to integrate neonatal clinical data with educational outcomes. The data analysis was performed between October 1, 2024, and October 31, 2025.

**Exposure:**

Gestational age and area-level socioeconomic deprivation.

**Main Outcomes and Measures:**

The main outcome was school readiness at age 5 years (as measured by the Early Years Foundation Stage Profile [EYFSP]) and attainment in reading, writing, mathematics, and science at age 6 to 7 years. For each outcome, prevalence of not meeting the expected level of attainment across indices of socioeconomic deprivation and GA stratified by 23 to 26 weeks and 27 to 31 weeks were calculated.

**Results:**

Of a total of 15 857 children included (2595 born at GA 23-26 weeks [16.3%] and 13 262 born at GA 27-31 weeks [83.7%]; 8449 boys [53.3%]), 8602 (56.6%) did not meet the school readiness level at age 5 years, and 7789 (51.8%) did not meet expected attainment at age 6 to 7 years for writing, 7216 (48.1%) for math, 6354 (41.9%) for reading, and 5449 (36.0%) for science. Children born at GA 23 to 24 weeks had a higher odds of not meeting expected school readiness levels compared with those born at 31 weeks (adjusted odds ratio [AOR], 2.86 [95% CI, 2.19-3.73]). Children born in areas with the most deprivation had a higher risk of underattainment compared with those in areas with the least deprivation (EYFSP: AOR, 1.27 [95% CI, 1.11-1.45]). In adjusted models, male sex and season of birth were associated with increased risk (EYFSP: AOR, 1.96 [95% CI, 1.82-2.11] and 2.64 [95% CI, 2.41-2.90], respectively) alongside several potentially modifiable risk factors, including smoking during pregnancy (AOR range from 1.19 [95% CI, 1.08-1.31] for math to 1.31 [95% CI, 1.19-1.44] for reading); exposure to postnatal corticosteroids (EYFSP: AOR, 1.37 [95% CI, 1.13-1.65]); severe acquired neonatal brain injuries, particularly periventricular leukomalacia (EYFSP: AOR, 3.05 [95% CI, 2.04-4.58]) and hydrocephalus (EYFSP: AOR, 2.47 [95% CI, 1.48-4.13]); comorbidities of preterm birth, including necrotizing enterocolitis (EYFSP: AOR, 1.60 [95% CI, 1.20-2.14], retinopathy of prematurity (EYFSP: AOR, 1.46 [95% CI, 1.10-1.93], and bronchopulmonary dysplasia (EYFSP: AOR, 1.30 [95% CI, 1.18-1.44]); and nutrition during neonatal care (EYFSP: AOR, 1.19 [95% CI, 1.07-1.33] and 1.51 [95% CI, 1.37-1.66] for mixed feeding and exclusive formula feeding, respectively, vs breastfeeding).

**Conclusions and Relevance:**

This cohort study of children born before GA 32 weeks found that preterm birth was associated with a substantial and persistent risk for educational underattainment across early school years, especially when combined with socioeconomic deprivation. Improving outcomes for children born preterm may require reducing social inequalities and minimizing comorbidities of preterm birth. Several modifiable early-life exposures offer practical targets for intervention, including maternal nonsmoking, appropriate corticosteroid use, and breastfeeding. Deferred school entry or targeted academic support may benefit children born very preterm, depending on birth season. These strategies may help parents, clinicians, educators, and policymakers improve long-term educational attainment for children born preterm.

## Key Points

**Question:**

Which early-life health and socioeconomic factors are associated with school readiness at age 5 years and educational attainment at age 6 to 7 years among children born very preterm?

**Findings:**

In this cohort study of 15 857 children born before gestational age 32 weeks, more than half did not meet expected levels of educational attainment at age 5 to 7 years. Low gestational age and social disadvantage showed dose-response associations with low attainment; neonatal brain injury, comorbidities of preterm birth, maternal smoking, lack of exposure to antenatal corticosteroids or exposure to postnatal corticosteroids, and nonexclusive breastfeeding were also associated with low attainment.

**Meaning:**

These findings suggest that improving the educational outcomes of very preterm children may require measures that mitigate social disadvantage and address specific, modifiable, early-life medical risks.

## Teaser

This cohort study evaluates which early-life health and socioeconomic factors are associated with school readiness and educational attainment among children aged 5 to 7 years born very preterm in the UK.

## Introduction

Globally, preterm birth affects 13.4 million pregnancies anually.^1^ Over the past 2 decades, the survival rate of children born preterm has improved, but 10% to 15% of children born very preterm (gestational age [GA] <32 weeks) develop cerebral palsy, and 30% to 50% develop intellectual or behavioral disabilities.^2^ Furthermore, preterm birth is more common among socioeconomically disadvantaged groups, reflecting inequalities in maternal health, environmental exposures, and access to antenatal care.^3^ Consequently, children who were born preterm face a double burden of biological immaturity and social disadvantage, with the latter influencing developmental outcomes through limited access to resources, greater exposure to stress, and reduced opportunities for cognitive stimulation.

However, some children born very preterm show typical neurodevelopment and cognition, indicating that low GA alone does not confer risk. We hypothesized that preterm birth–associated risk factors, which are biological, psychosocial, and social or infrastructural and commonly co-occur with preterm birth, influence susceptibility to injury and dysmaturation and may explain differences in school-age attainment. Such factors, for instance alterations in the perinatal stress environment,^4,5^ systemic inflammation,^6,7^ suboptimal neonatal nutrition,^8^ complications of preterm birth (eg, bronchopulmonary dysplasia, necrotizing enterocolitis),^9^ and social inequalities, can affect parent or child or be shared.^3,10,11^

Cohort studies and meta-analyses consistently have revealed a dose-dependent association between low GA and child cognition or proxies of educational attainment, such as special educational needs, but their designs have left uncertainty about the maternal and neonatal variables associated with educational performance and the role of social disadvantage.^12,13,14,15,16,17,18^ Furthermore, older cohorts do not reflect the substantial changes in clinical complexity, patient outcomes, and demographic changes of mothers that have taken place in recent years or current health, social, and education policy contexts. Consequently, there are knowledge gaps about the association of preterm birth risk factors in the modern era of perinatal care with school readiness and early-years educational attainment, which, in turn, shape the support a child will receive, and the relative importance of deprivation in shaping this association. Understanding the extent of educational underachievement among children aged 5 to 7 years who were born preterm and clarifying the relative contributions of perinatal medical factors and social disadvantage are essential. This knowledge is needed to design evidence-based strategies that promote brain health after preterm birth and to improve support during the transition to school, when cognitive demands increase and life course trajectories are potentially modifiable.

The National Neonatal Research Database (NNRD) contains data on all UK neonatal admissions from 2007 to the present.^19^ The National Pupil Database (NPD) is an administrative dataset of educational outcomes collected and maintained by the Department for Education of all state-funded pupils in England.^20^ We created a novel linkage between the NNRD and the NPD^21^ to quantify the proportion of preterm children not meeting expected levels in statutory assessments and tested the hypothesis that specific preterm birth risk factors modify the association between GA and outcomes for school readiness and educational attainment at age 5 to 7 years among children in England.

## Methods

This retrospective cohort study linked data from the NNRD^10^ and NPD^11,21^ (dataset details provided in the eMethods in Supplement 1). The cohort comprised children born and cared for in a neonatal unit in England between September 1, 2008, and August 31, 2012, with a recorded GA of less than 32 completed weeks, who survived to discharge, and who had a linked neonatal and education record. The study epoch was chosen to include children old enough to be eligible for assessments at 5 to 7 years. Infants with congenital malformations or missing data on GA, sex, or place of birth were excluded. Research ethical and regulatory approval was granted to use deidentified data as part of the neoWONDER study, including Confidentiality Advisory Group approval to use identifiers for linkage purposes without informed consent.^21^ No neonatal unit opt-out requests were received. This study followed the Strengthening the Reporting of Observational Studies in Epidemiology (STROBE) reporting guideline for cohort studies.

### Definitions of Variables

All outcomes were binary indicators of whether a child met expected educational levels at ages 5, 6, and 7 years, ascertained from statutory teacher-assessed outcomes in England. At the end of age 5 reception (akin to preschool), teachers assess 7 domains of the Early Years Foundation Stage Profile (EYFSP): communication and language; physical development; personal, social, and emotional development; literacy; mathematics; understanding the world; and expressive arts and design. A good level of development was defined as achieving at least the expected level in communication and language; physical development; personal, social, and emotional development; literacy; and mathematics.^22^ The EYFSP was used as a measure of school readiness because these domains reflect skills important for early learning.^23^

During year 1, teachers assess key stage 1 (KS1) phonics at age 6 years using a 40-word test to assess reading ability. Pupils who score 32 or more out of 40 meet the expected level.^24^ At the end of KS1 assessments at age 7 years, binary variables were derived to denote whether children met the expected level in national tests of reading, writing, mathematics, and science. For KS1 assessments, the outcome was expressed as not meeting the expected level at each time point because it indicates children who are likely to require additional support.^16,25^

### Exposures

Gestational age was defined as completed weeks; for descriptive analyses, we report outcomes stratified by GA groups: 23 to 26 weeks and 27 to 31 weeks.^26^ The Index of Multiple Deprivation (IMD), a UK area-level composite measure of socioeconomic deprivation, was derived from the maternal postal code recorded in the NNRD at birth.^27^ It combines 7 domains: income, employment, education, health, crime, barriers to housing and services, and living environment. Maternal postal codes were linked to the UK Office for National Statistics Lower Super Output Areas to assign IMD scores and grouped into national deciles (1, most deprived; 10, least deprived).

### Covariates

Preterm birth risk factors included maternal smoking, age, race and ethnicity, gestational diabetes mellitus, and hypertensive disorders. Neonatal characteristics included sex, birth month, birth weight, multiplicity, mode of delivery, comorbidities of preterm birth (retinopathy of prematurity, necrotizing enterocolitis, sepsis, bronchopulmonary dysplasia), brain injuries (cystic periventricular leukomalacia, all-grade intraventricular hemorrhage, hydrocephalus, porencephalic cyst), exposure to antenatal and postnatal corticosteroids, and nutrition at discharge (definitions provided in eTable 1 in Supplement 1). Maternal race and ethnicity was self-reported and grouped according to the 2011 National Census categories (Asian, Black, White, multiracial, other [Arab or Chinese]).^28^ Race and ethnicity were included because they may influence educational outcomes through social, environmental, and health care–related factors.

Socioeconomic and demographic variables from the NPD included race and ethnicity, major language group, and the Income Deprivation Affecting Children Index (IDACI) at age 5 years, which ranks neighborhoods according to the proportion of children younger than 16 years living in low-income households, expressed as deciles.^27^ Eligibility for free school meals at age 5 years was a family-level proxy for household income.

### Statistical Analysis

The data were analyzed between October 1, 2024, and October 31, 2025. We report characteristics for children with and without a linked education record (Table), presenting frequencies with percentages for categorical variables and medians with interquartile ranges for continuous variables. For each outcome, we plotted the prevalence (95% CI) of not meeting the expected level of attainment across IMD, IDACI, and free school meals stratified by GA 23 to 26 weeks and 27 to 31 weeks. Cohort outcomes were compared with Department for Education national data for the same epoch.^22,24^

**Table.** Characteristics of Children With and Without a Linked NNRD-NPD Record

Characteristic	Children, No. (%)		
	Not linked (n = 1029)	Linked (n = 15 857)	Total (N = 16 886)
**Child**			
Gestational age, completed wk			
23-24	44 (4.3)	651 (4.1)	677 (4.1)
25	49 (4.8)	748 (4.7)	797 (4.7)
26	80 (7.8)	1196 (7.5)	1276 (7.6)
27	105 (10.2)	1546 (9.7)	1651 (9.8)
28	125 (12.1)	2091 (13.2)	2216 (13.1)
29	160 (15.5)	2399 (15.1)	2559 (15.2)
30	215 (20.9)	3068 (19.3)	3283 (19.4)
31	251 (24.4)	4158 (26.2)	4409 (26.1)
Birth weight *z* score, median (IQR)	−0.21 (−0.82 to 0.30)	−0.21 (−0.81 to 0.31)	−0.21 (−0.81 to 0.31)
Small for gestational age	142 (13.8)	2135 (13.5)	2277 (13.5)
Birth multiplicity			
Singleton	715 (69.5)	11 582 (73.1)	12 297 (72.8)
Multiple	314 (30.5)	4270 (26.9)	4584 (27.2)
Sex assigned at birth			
Female	460 (44.7)	7408 (46.7)	7868 (46.6)
Male	569 (55.3)	8449 (53.3)	9018 (53.4)
**Socioeconomic status**			
IMD decile at birth			
1 (Most deprived)	104 (10.9)	2847 (18.5)	2951 (18.0)
2	118 (12.3)	2285 (14.8)	2403 (14.7)
3	105 (11.0)	1939 (12.6)	2044 (12.5)
4	103 (10.8)	1597 (10.4)	1700 (10.4)
5	70 (7.3)	1520 (9.9)	1590 (9.7)
6	98 (10.2)	1241 (8.0)	1339 (8.2)
7	85 (8.9)	1144 (7.4)	1229 (7.5)
8	95 (9.9)	1045 (6.8)	1140 (7.0)
9	96 (10.0)	916 (5.9)	1012 (6.2)
10 (Least deprived)	83 (8.7)	885 (5.7)	968 (5.9)
**Maternal**			
Age at delivery, median (IQR), y	33 (28 to 36)	30 (25 to 34)	30 (25 to 35)
Race and ethnicity			
Asian	148 (15.2)	1612 (10.7)	1760 (11.0)
Black	98 (10.1)	1362 (9.1)	1460 (9.1)
White	661 (67.9)	11 560 (76.9)	12 221 (76.3)
Multiracial	13 (1.3)	246 (1.6)	259 (1.6)
Other^a^	53 (5.4)	254 (1.7)	307 (1.9)
Smoking while pregnant	79 (7.7)	2906 (18.3)	2985 (17.7)
Diabetes	24 (2.3)	457 (2.9)	481 (2.8)
Hypertensive disorders of pregnancy	140 (13.6)	2037 (12.8)	2177 (12.9)
Interventions			
Completed course of antenatal corticosteroids	701 (73.2)	10 458 (70.0)	11 159 (70.2)
Postnatal corticosteroids	58 (5.8)	1095 (7.1)	1153 (7.0)
**Neonatal morbidities**			
Sepsis	49 (4.8)	785 (5.0)	834 (4.9)
Necrotizing enterocolitis	17 (1.7)	362 (2.3)	379 (2.2)
Retinopathy of prematurity	18 (1.7)	462 (2.9)	480 (2.8)
Bronchopulmonary dysplasia	264 (26.0)	4195 (26.9)	4459 (26.9)
**Neonatal brain injuries**			
Cystic periventricular leukomalacia	<10 (0.9)	NR (1.6)^b^	NR (1.6)^b^
Intraventricular hemorrhage			
None	902 (87.7)	13 775 (86.9)	14 677 (86.9)
Grade I-II	91 (8.8)	1648 (10.4)	1739 (10.3)
Grade III-IV	36 (3.5)	434 (2.7)	470 (2.8)
Porencephalic cyst	15 (1.5)	202 (1.3)	217 (1.3)
Hydrocephalus	<10 (0.5)	NR (1.1)^b^	NR (1.0)^b^
**Nutrition at discharge**			
Enteral milk at discharge			
Breast	458 (47.6)	4257 (28.8)	4715 (29.9)
Formula	204 (21.2)	5946 (40.2)	6150 (39.0)
Other	57 (5.9)	1497 (10.1)	1554 (9.9)
Mixed	243 (25.3)	3091 (20.9)	3334 (21.2)

Abbreviations: IMD, Index of Multiple Deprivation; NNRD, National Neonatal Research Database; NPD, National Pupil Database; NR, not reported.

^a^ Included Arab and Chinese.

^b^ Frequencies were suppressed to minimize risk of secondary disclosure when cell counts described less than 10 individuals.

Missingness was low (0.8%) and addressed using multiple imputation by chained equations (eMethods and eTable 2 in Supplement 1). We fitted cross-sectional logistic regression models using generalized estimating equations (GEEs) with an exchangeable correlation structure to account for within-mother correlation among multiple births, assuming equal correlation between siblings.^29^ Models used a binomial distribution with a logit link and robust standard errors. Multicollinearity was assessed using the variance inflation factor (threshold <5). Children with complete outcome data and maternal identifiers were included in the GEE models.

The IMD quintiles were used in 3 models to highlight broader gradients of deprivation in line with previous studies: (1) crude associations; (2) GA and IMD interaction models, assessed using significance testing (at the *P* < .05 level) of the interaction term and probability plots of outcomes at mean values of covariates values stratified by GA across levels of IMD; and (3) fully adjusted models, including all described covariates. To avoid collinearity among race and ethnicity, language, and socioeconomic variables (eFigure 1 in Supplement 1), primary models included IMD, free school meal eligibility, and language.

Sensitivity analyses substituted race and ethnicity for language. Results are reported as odds ratios (ORs) with 95% CIs. Confidence intervals not crossing 1 were considered statistically significant at 5%. Model performance was evaluated as described in the eMethods in Supplement 1. Analyses were conducted using R, version 4.5.0 (R Foundation for Statistical Computing) and Stata, version 18.0 (StataCorp LLC).

## Results

### Study Population

Of 23 862 infants in the NNRD, 17 245 (72.3%) were identified by NHS England for linkage (of whom 359 died before school age), and 6617 (27.7%) were not identified due to missing or incomplete NHS numbers. Of those identified for linkage and alive at school entry, 15 857 of 16 886 (93.9%) had an education record in the NPD (2595 born at GA 23-26 weeks [16.3%] and 13 262 born at GA 27-31 weeks [83.7%]; 7408 female [44.7%] and 8449 male [53.3%]; 4270 part of a multiple birth [26.9%]; 1612 born to Asian [10.7%], 1362 to Black [9.1%], 11 560 to White [76.9%], and 246 to multiracial [1.6%] mothers and 254 [1.7%] to mothers identifying as other race and ethnicity) (eFigure 2 in Supplement 1; Table); 1029 children (6.0%) did not have an educational record. There were no major differences between groups with and without sufficient linkage identifiers (eTable 3 in Supplement 1). There were no differences in the distribution of GA, sex, or neonatal morbidities between linked and nonlinked groups; however, the linked group included more children whose mothers identified as White (11 560 [76.9%] compared with 661 [67.9%]) and were from the most deprived IMD decile (2847 [18.5%] vs 104 [10.9%]).

### Trends in Educational Attainment of the Cohort and Comparison With the Whole Population

eTable 4 in Supplement 1 presents the annual proportion of the cohort not meeting expected educational levels, and eFigure 3 in Supplement 1 summarizes these alongside national population results. At age 5 to 6 years, children born preterm underperformed compared with the national average (EYFSP: 8602 children [56.6%] vs 232 768 of 658 899 children [35.4%]; phonics: 3226 [20.7%] vs 56 749 of 649 287 children [8.7%]). At age 7 years, approximately one-half did not meet expected levels in writing (7789 children [51.8%]) and math (7216 children [48.1%]), and approximately 40% did not meet expected levels in reading (6354 children [41.9%]) and science (5449 children [36.0%]). The differences from national averages were 21.1 percentage points for EYFSP, 11.9 percentage points for phonics, 16.9 percentage points for KS1 reading, 20.0 percentage points for KS1 writing, 23.1 percentage points for KS1 math, and 18.5 percentage points for KS1 science (eFigure 3 in Supplement 1).^22,24^ Children born before GA 32 weeks constituted 1.2% of the national comparator data.^30^

### Associations Between GA and Educational Attainment

**Figure 1.**
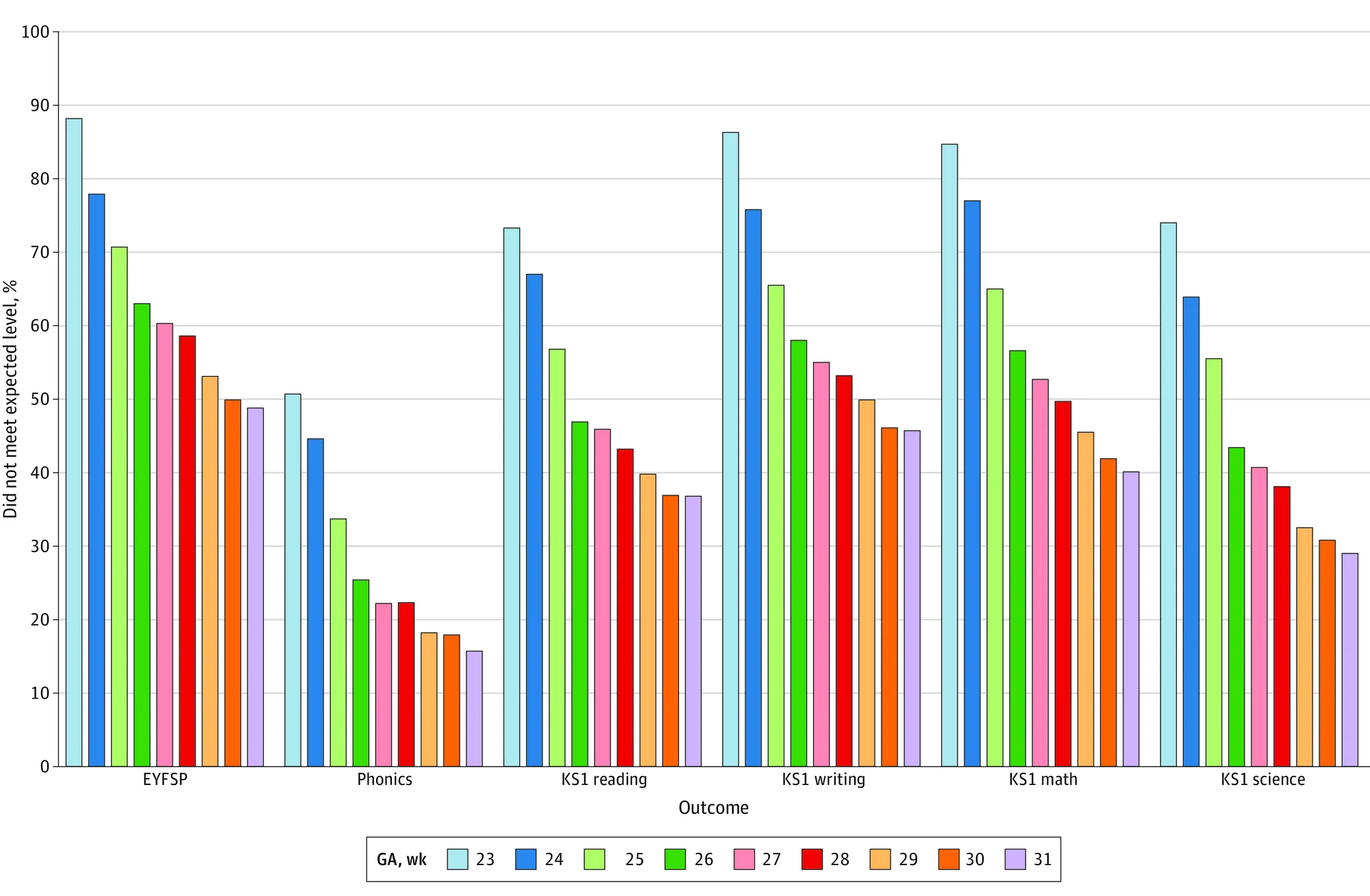
Bar Graph of the Distribution of Children Not Meeting Expected Levels by Outcome and Gestational Age (GA) Proportion in the overall cohort who did not meet expected level: Early Years Foundation Stage Profile (EYFSP), 8602 of 15 528 (55.9%); phonics, 3226 of 15 504 (20.8%); key stage 1 (KS1) reading, 6354 of 15 141 (42.0%); KS1 writing, 7789 of 15 141 (51.4%); KS1 math, 7216 of 15 140 (47.7%); KS1 science 5449 of 15 141 (36.0%).

There was a dose-response association between GA and educational attainment. Each decreasing week of gestation from 31 weeks was associated with a higher proportion of children not achieving expected levels across all outcomes (Figure 1; eTable 5 in Supplement 1). For children born at GA 23 weeks, 120 of 136 (88.2%) did not meet expected levels for EYFSP and 96 of 131 (73.3%), 111 of 131 (84.7%), 97 of 131 (74.0%), and 113 of 131 (86.3%) did not meet expected levels for the KS1 reading, math, science, and writing domains, respectively, compared with those born at GA 31 weeks (EYFSP, 1992 of 4083 [48.8%]; KS1 reading, 1472 of 3996 [36.8%]; KS1 math, 1601 of 3995 [40.1%]; KS1 science, 1157 of 3996 [29.0%]; KS1 writing, 1828 of 3996 [45.7%]).

### Associations Between Socioeconomic Status and Educational Attainment

**Figure 2.**
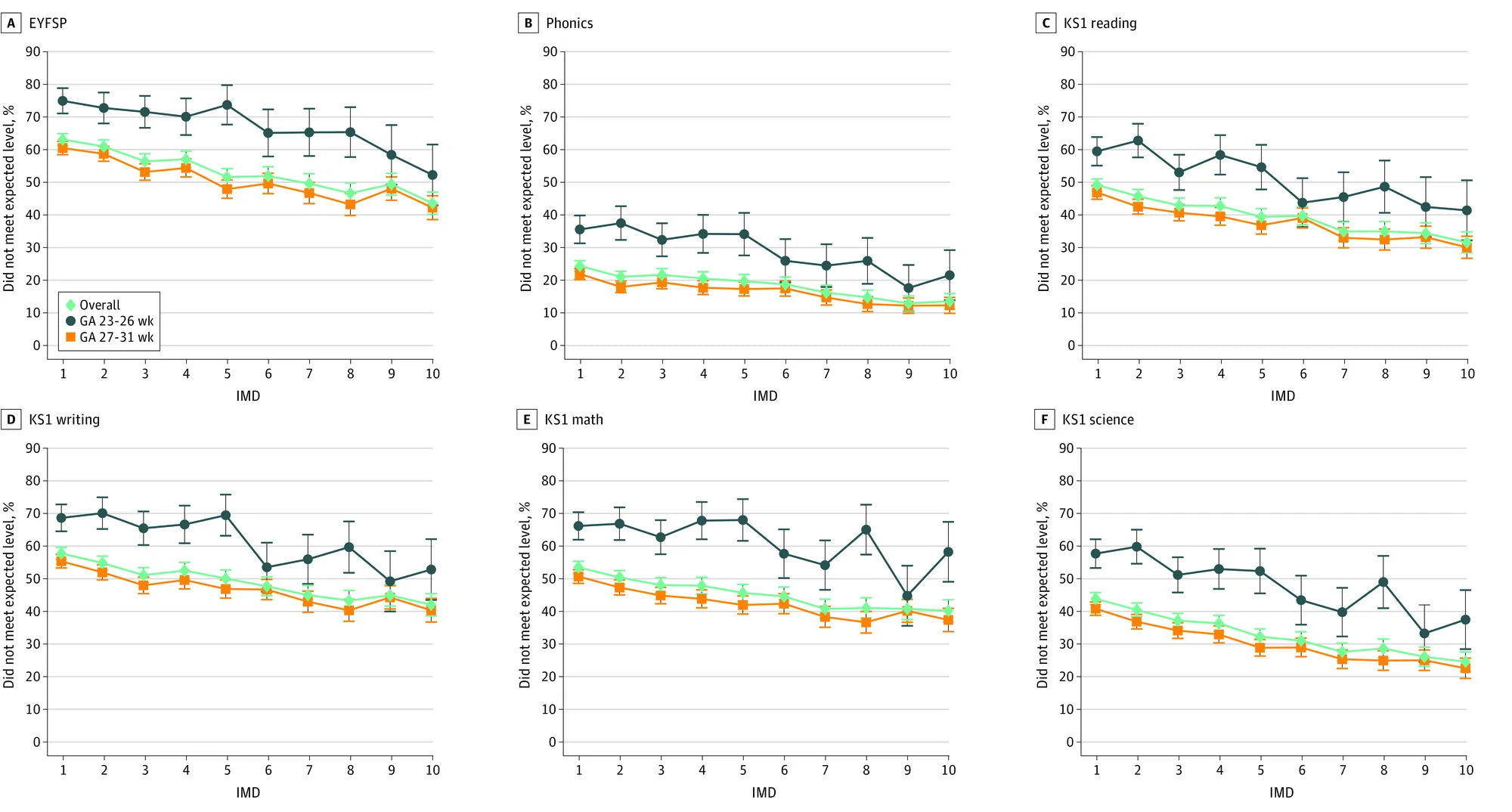
Dot Plots of the Proportion of Children Who Did Not Meet the Expected Level in Outcomes by Socioeconomic Status at Birth (Index of Multiple Deprivation [IMD]), Stratified by Gestational Age (GA) Category and the Overall Cohort Trend tested using the Cochran-Armitage test. *P* < .001 for every line. Error bars indicate the 95% CI. Index of Multiple Deprivation decile 1 indicates the most deprivation and decile 10, the least deprivation. EYFSP indicates Early Years Foundation Stage Profile; KS1, key stage 1.

The proportion of children who did not meet expected levels declined progressively with higher socioeconomic status at birth (Figure 2). The gradients across IMD levels were parallel, indicating similar outcomes associated with deprivation across GA groups. Among children born in the least deprived IMD decile, 58 of 111 (52.3%) born at GA 23 to 26 weeks and 300 of 710 (42.3%) born at 27 to 31 weeks did not achieve the expected level at EYFSP. For the most deprived decile, the corresponding proportions were higher at 364 of 486 (74.9%) and 1336 of 2209 (60.5%), respectively. These trends were consistent for free school meals and IDACI at 5 years (eFigures 4 and 5 in Supplement 1).

### Longitudinal Individual Educational Attainment Trajectories

**Figure 3.**
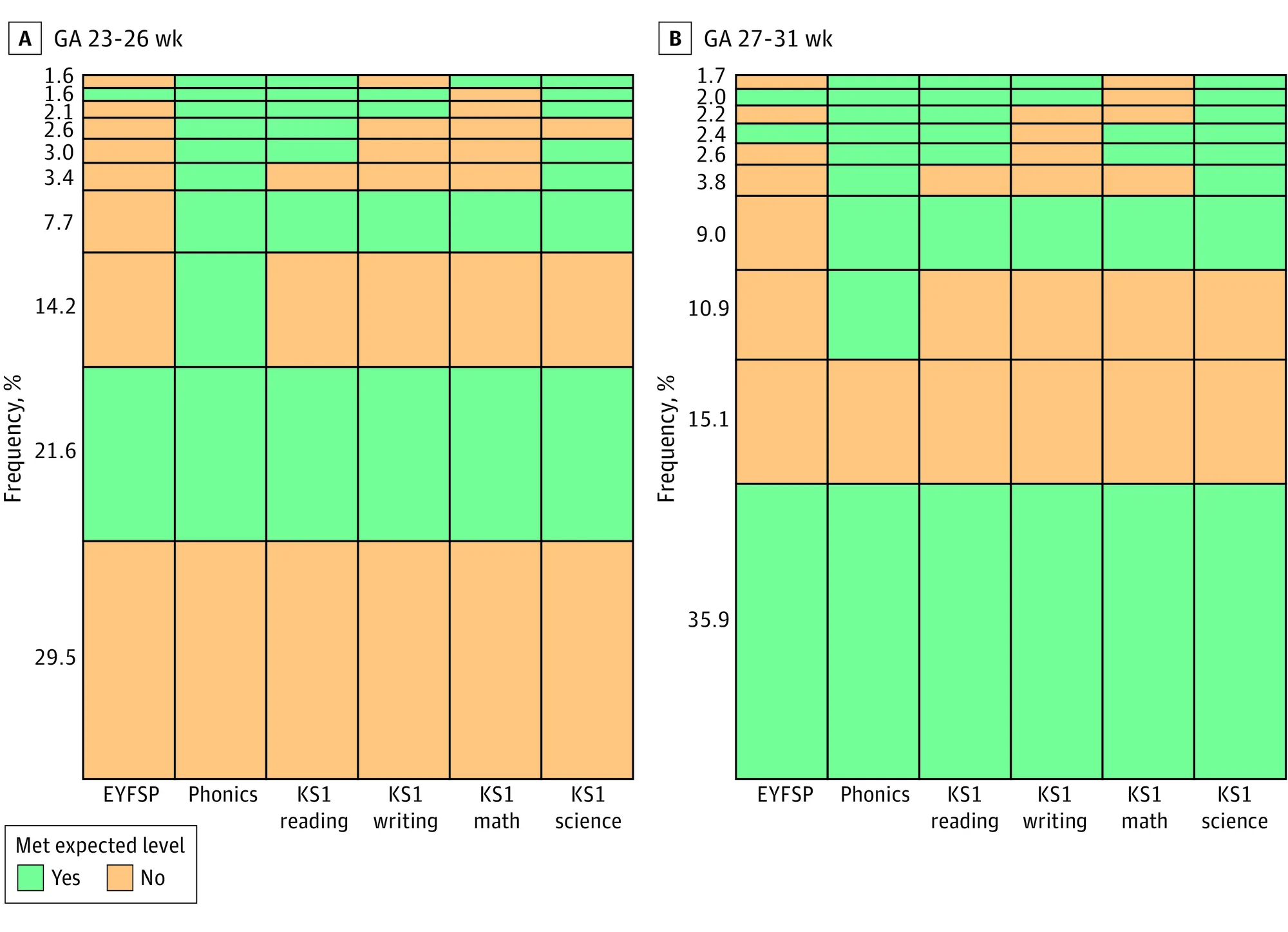
Sequence Frequency Plots of the Top 10 Longitudinal Outcome Patterns Across Age 5 to 7 Years, by Gestational Age (GA) Groups The y-axis represents the cumulative percentage of longitudinal sequences, and horizontal bar widths are proportional to their frequencies. Rows are ordered by frequency of the pattern. EYFSP indicates Early Years Foundation Stage Profile; KS1, key stage 1.

Figure 3 shows the 10 most common educational attainment profiles. Persistent underperformance in all outcomes was the leading pattern among children born at GA 23 to 26 weeks (29.5%), who were more likely to miss expected levels in 5 or more outcomes than those born at GA 27 to 31 weeks (43.7% vs 26.0%). For children born at GA 27 to 31 weeks, the most frequent pattern was meeting expectations in all outcomes (35.9% vs 21.6% at GA 23-26 weeks).

### Factors Associated With Educational Attainment in Adjusted Models

#### GA and Other Infant Characteristics

**Figure 4.**
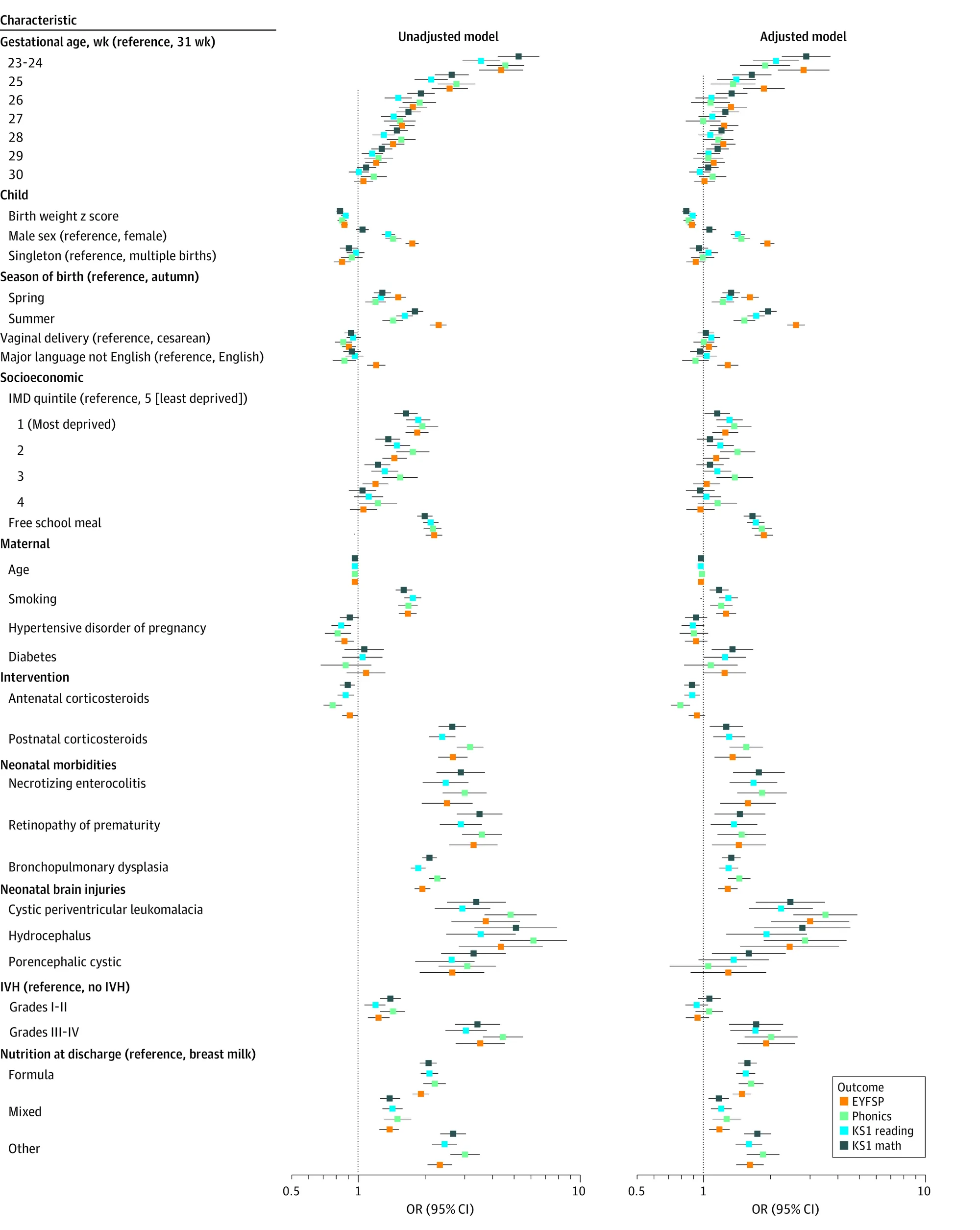
Forest Plots of Unadjusted and Adjusted Associations of Socioeconomic and Preterm Birth Risk Factors With Educational Outcomes Odds ratios (ORs) and 95% CIs derived from generalized estimating equation regression models for not meeting the expected level in Early Years Foundation Stage Profile (EYFSP), phonics, and key stage 1 (KS1) reading or math. Numeric values of ORs are provided in eTable 6 in Supplement 1. IMD indicates Index of Multiple Deprivation; IVH, intraventricular hemorrhage.

eTable 6 in Supplement 1 and Figure 4 present the ORs and 95% CIs from the unadjusted and fully adjusted GEE regression models for EYFSP, phonics, and KS1 reading and math domains. There were strong correlations across domains for KS1 7-year outcomes (*r* >0.8) (eFigure 1 in Supplement 1); the results for KS1 writing and science are presented in eTable 7 and eFigure 6 in Supplement 1.

A total of 14 129 children with complete outcome data and a maternal identifier were included in the models. The dose-response association between GA and attainment persisted after adjustment, particularly for the most preterm infants, though attenuated. Children born at GA 23 to 24 weeks were more likely to not achieve expected EYFSP levels compared with those born at GA 31 weeks (adjusted odds ratio [AOR], 2.86 [95% CI, 2.19-3.73]) (eTable 6 in Supplement 1). Higher birth weight and female sex were protective, although sex differences narrowed by age 7 years, especially for math (EYFSP: AOR, 1.96 [95% CI, 1.82-2.11]; math: AOR, 1.07 [95% CI, 1.00-1.15]). Children born in the summer who enter school a year earlier than those born in the autumn were more likely to underachieve at EYFSP (AOR, 2.64 [95% CI, 2.41-2.90]).

#### Maternal Characteristics

Smoking increased the odds of underattainment (AOR range from 1.19 [95% CI, 1.08-1.31] for math to 1.31 [95% CI, 1.19-1.44] for reading) (eTable 6 in Supplement 1). Increasing maternal age was protective, with each additional year decreasing the odds of underattainment (AOR range from 0.98 [95% CI, 0.98-0.99] for reading to 1.00 [95% CI, 0.99-1.00] for phonics). The association of maternal diabetes increased after adjustment (AOR range from 1.26 [95% CI, 1.01-1.57] for EYFSP to 1.36 [95% CI, 1.10-1.69] for math); however, hypertensive disorders had no association with outcomes.

#### Interventions in the Perinatal Period

A complete course of antenatal corticosteroids was protective across all domains, whereas postnatal corticosteroids were consistently associated with elevated odds of underattainment (EYFSP: AOR, 1.37 [95% CI, 1.13-1.65]; math: AOR, 1.28 [95% CI, 1.08-1.52]) (eTable 6 in Supplement 1). Lack of exclusive breastfeeding was associated with poorer outcomes. Mixed feeding had a lower association than exclusive formula feeding (EYFSP: AOR, 1.19 [95% CI, 1.07-1.33] vs 1.51 [95% CI, 1.37-1.66], respectively).

#### Preterm Complications and Comorbidities

Neonatal brain injuries were associated with the highest odds of underattainment, particularly periventricular leukomalacia (EYFSP: AOR, 3.05 [95% CI, 2.04-4.58]) and hydrocephalus (EYFSP: AOR, 2.47 [95% CI, 1.48-4.13]) (eTable 6 in Supplement 1). Of all covariates, periventricular leukomalacia showed the least attenuation after adjustment and had an effect size on par with being born at GA 23 to 24 weeks. Porencephalic cysts were associated only with KS1 math outcomes (AOR, 1.62 [95% CI, 1.10-2.37]). Necrotizing enterocolitis, treated retinopathy of prematurity, and bronchopulmonary dysplasia were associated with a higher odds of poor outcomes (EYFSP: AOR, 1.60 [95% CI, 1.20-2.14], 1.46 [95% CI, 1.10-1.93], and 1.30 [95% CI, 1.18-1.44], respectively), while sepsis showed no association.

#### Socioeconomic Exposures

Socioeconomic disadvantage was negatively associated with attainment at all ages. In adjusted models, children born in the most deprived IMD quintile were more likely to underachieve than those in the least deprived quintile (EYFSP: AOR, 1.27 [95% CI, 1.11-1.45]), and children eligible for free school meals were more likely to have poor outcomes (EYFSP: AOR, 1.89 [95% CI, 1.72-2.08]), a magnitude comparable to severe intraventricular hemorrhage (grade III-IV) (EYSFP: AOR, 1.94 [95% CI, 1.44-2.61]) (eTable 6 in Supplement 1). Children whose primary language was not English were more likely to underachieve at EYFSP (AOR, 1.30 [95% CI, 1.17-1.45]), though this difference was not present at KS1.

Findings were consistent for KS1 science and writing (eFigure 6 and eTable 7 in Supplement 1). There was no statistical interaction between deprivation and GA; the probability of poor attainment increased steadily with higher deprivation in all GA groups, indicating similar negative associations regardless of GA (eFigure 7 in Supplement 1).

Models showed low multicollinearity (variance inflation factor, 1.28) and moderate discrimination (area under the curve ≈ 0.7) and classification performance (balanced accuracy of 0.63-0.66). Sensitivity analyses substituting language with race and ethnicity showed no consistent associations with race and ethnicity and IMD, and free school meal coefficients remained stable (eTable 8 in Supplement 1).

## Discussion

This large, population-based cohort study of preterm infants born in England before GA 32 week between 2008 and 2012 found significantly elevated risks of not being ready for school at age 5 years and of not meeting expected educational attainment levels at age 6 and 7 years. There was a dose-response association in which only one-fifth of children born at GA 23 to 26 weeks and one-third born at GA 27 to 31 weeks met expected levels of attainment at all time points. The data highlight the additive association of socioeconomic deprivation with educational outcomes of children born very preterm. Those born in the most deprived quintile of neighborhoods were 20% to 40% more likely not to meet expected levels than those born in the least deprived quintile, even after accounting for low GA and medical complications. Free school meal eligibility at age 5 years was associated with an almost doubled risk of underattainment, an effect size comparable to that of severe neonatal brain injury. Index of Multiple Deprivation, IDACI, and free school meals as proxies for social and family-level contexts may influence development through access to resources, health behaviors, and caregiving environments. Interventions may need to operate at structural (eg, reducing child poverty), family (eg, supporting parent-infant interaction), and area (eg, access to high-quality early-years education) levels, particularly for children born very preterm.^13,31^ Causal inference is limited, and further research is needed to clarify mechanisms and identify effective interventions.

Our findings corroborate education research showing that deprivation is associated with preschool neurodevelopment^32,33^ and cognitive neuroscience research showing that inequalities are associated with differences in brain structure and connectivity, particularly in regions that support language, executive function, and memory, among preterm children.^10,34^ The data suggest that social deprivation and preterm birth constitute a double burden. These negative associations may be modifiable through family-level interventions and social policy designed to alleviate deprivation, particularly as one-third of children in the UK live in very-low-resourced areas.^35^ Continued clinical and research efforts are needed to reduce severe neonatal brain injuries, necrotizing enterocolitis, bronchopulmonary dysplasia, and retinopathy of prematurity, which were associated with low educational attainment. While postnatal corticosteroids were associated with low attainment, further work is required to explore whether this is a proxy of illness severity or a direct effect on the developing brain.

Although some of the preterm birth risk factors are immutable (GA, male sex), others are potentially modifiable with targeted interventions, such as increased breastfeeding rates, improved antenatal corticosteroid use, and reduced maternal smoking. The finding that children born in the summer were at higher risk of underattainment is consistent with prior literature, although the effects of delayed school entry may vary by socioeconomic context.^13,31^ This finding has implications for school entry policies for very preterm children and requires further study. Studies are required to determine whether very preterm boys and children growing up in communities with high deprivation require additional support. Ensuring that children born preterm with additional risk factors are supported for school readiness might confer life course and societal benefits, including higher educational achievement and improved employability and income.^36,37^

This study included the use of contemporary, socioeconomically, and racially and ethnically diverse population-level datasets through a novel linkage between the NNRD and NPD, the first to our knowledge to combine these national resources. This linkage enabled integration of maternal and neonatal clinical data with actual educational outcomes at school entry. The scale and depth of the data allowed granular characterization of how early-life clinical factors and social disadvantage jointly contribute to educational underattainment among children born before GA 32 weeks.

Socioeconomic status was triangulated using complementary measures, including IMD, IDACI, and free school meal eligibility, reflecting both neighborhood-level and family-level disadvantage. The large cohort size substantially exceeded previous linkage studies and enabled robust estimation of associations across a wide range of perinatal and social factors.^16,25^ This study extends existing evidence by examining contemporary statutory educational outcomes, including school readiness at age 5 years and attainment at age 6 to 7 years across multiple domains. Unlike earlier cohorts based on births from the 1980s to 1990s or proxy outcomes (eg, special educational needs), this analysis reflects modern perinatal care and current policy contexts, allowing identification of early-life determinants of education-relevant outcomes in today’s population. Population-level monitoring of these outcomes is important for evaluating temporal trends and detecting the effects of broad neonatal strategies, thereby providing valuable surveillance of the long-term educational consequences of very preterm birth.

### Limitations

This study had several limitations, including the 27.7% of eligible children not linked due to missing or incomplete NHS numbers. Among those identified, 1029 children (6.1%) lacked an education record, possibly reflecting home schooling, private or hospital schooling, or migration. As the NPD does not capture children outside the state-funded education system, we could not characterize this subgroup, which may have introduced selection bias, although the direction is uncertain. Our adjusted models achieved a moderately discriminative area under the curve of 0.7, and omitted variable bias may have resulted from unavailable data on factors underlying early development, including parental education, parent-infant interactions, health behaviors, nutrition, and access to services and educational resources.^23^ Individual-level maternal (or parental) educational attainment, an important determinant of child outcomes, was unavailable, which linkage to area-level educational indicators (eg, census data) may partially mitigate in future research. We could not adjust for preexisting neurodevelopmental impairment, which may have confounded school-age outcomes.^32^ The absence of a control group of children born at term may have limited power to detect interactions between GA and socioeconomic deprivation. Findings may not fully generalize to low- and middle-income country settings; however, maternal health behaviors, antenatal corticosteroid use, breast milk, and reduction of comorbidities may be advantageous.^38^

## Conclusions

This cohort study of children born before GA 32 weeks found that approximately one-half did not meet expected educational attainment levels at age 5 to 7 years. Low GA, socioeconomic deprivation, comorbidities, and several modifiable early-life exposures were identified. Multisectoral policies and research that reduce deprivation alongside improving maternal health, supporting caregiving environments, minimizing comorbidities of preterm birth, and targeting educational support may improve school performance and reduce life course impacts of being born prematurely.^23^ The identified early-life factors may prompt research into the biological processes that embed preterm birth and social inequalities in child development. We show the feasibility of linking neonatal health and education data, with parents supporting such a linkage.^21^ Continued follow-up could enable evaluation of interventions that reduce child deprivation.

## Notes

Supplement 1.
eMethods.
**eTable 1.** Description of Variables **eTable 2.** Distribution of Missing Data in Covariates **eTable 3.** Characteristics of Children With and Without Sufficient Personal Identifiers for Linkage **eTable 4.** Distribution of Children in the Preterm Cohort Who Did or Did Not Meet Expected Levels in Each Outcome by Birth Year **eTable 5.** Distribution of Children in the Preterm Cohort Who Did Not Meet Expected Levels in Each Outcome by Gestational Age (Birth Years 2008-2009 to 2011-2012) **eTable 6.** Unadjusted and Adjusted GEE Regression Models for the Association Between Socioeconomic and Preterm Birth Factors With Educational Outcomes for Children Born 2008 or 2009 to 2011 or 2012 in England **eTable 7.** Unadjusted and Adjusted GEE Regression Models for the Association Between Socioeconomic and Preterm Birth Factors With Educational Outcomes (KS1 Writing and Science) for Children Born 2008 or 2009 to 2011 or 2012 in England **eTable 8.** Sensitivity Analysis Substituting Child’s Language for Ethnicity: Fully Adjusted GEE Regression Models for the Association of Socioeconomic and Preterm Birth Factors With Educational Outcomes for Children Born 2008 or 2009 to 2011 or 2012 in England **eFigure 1.** Heat Map of Correlations Between Outcomes and Covariates **eFigure 2.** Participant Flow of Cohort Included in the Study Born Between 2008 or 2009 and 2011 or 2012 in England **eFigure 3.** Distribution of Children Who Did Not Meet Expected Levels for Each Outcome in the Cohort and the Whole National Population **eFigure 4.** Proportion of Children Who Did Not Meet the Expected Level in Outcomes by Income Deprivation Affecting Children Index (IDACI) at Age 5 Years, Stratified by Gestational Age Category (23-26 Weeks and 27-31 Weeks) and the Overall Cohort **eFigure 5.** Proportion of Children Who Did Not Meet the Expected Level in Outcomes by Free School Meal Eligibility at Age 5 Years, Stratified by Gestational Age Category (23-26 Weeks and 27-31 Weeks) and the Overall Cohort **eFigure 6.** Forest Plot Showing the Unadjusted and Adjusted Associations Between Socioeconomic and Preterm Birth Factors With Educational Outcomes (Not Meeting the Expected Level in KS1 Writing or KS1 Science) **eFigure 7.** Estimated Probability of Not Meeting Expected Levels in Educational Outcomes Across Index of Multiple Deprivation (IMD) at Birth, Stratified by Gestational Age
eReferences.

Supplement 2.
Data Sharing Statement
