# Error in Key Points and Figure 4

**DOI:** 10.1001/jamanetworkopen.2026.33687

**Published:** 2026-08-19

In the Original Investigation titled “Health and Socioeconomic Factors in School Readiness and Achievement Among Children Born Very Preterm,”^1^ published July 14, 2026, the Key Points Findings were revised to clarify that nonexclusive breastfeeding was associated with low educational attainment, and the order of the subcategory labels for intraventricular hemorrhage in Figure 4 was reversed. This article has been corrected.^1^

## References

[1] Haider S, Tsanas A, Batty GD, . Health and socioeconomic factors in school readiness and achievement among children born very preterm. JAMA Netw Open. 2026;9(7):e2623068. doi:10.1001/jamanetworkopen.2026.23068 42446880 PMC13370314

