# Supplementary material for: Health and Socioeconomic Factors in School Readiness and Achievement Among Children Born Very Preterm
Source: JAMA Netw Open. 2026 Jul 14;9(7):e2623068. doi: 10.1001/jamanetworkopen.2026.23068 (PMC13370314; doi:10.1001/jamanetworkopen.2026.23068)
Supplement: Supplement 1. — eMethods. eTable 1. Description of Variables eTable 2. Distribution of Missing Data in Covariates eTable 3. Characteristics of Children With and Without Sufficient Personal Identifiers for Linkage eTable 4. Distribution of Children in the Preterm Cohort Who Did or Did Not Meet Expected Levels in Each Outcome by Birth Year eTable 5. Distribution of Children in the Preterm Cohort Who Did Not Meet Expected Levels in Each Outcome by Gestational Age (Birth Years 2008-2009 to 2011-2012) eTable 6. Unadjusted and Adjusted GEE Regression Models for the Association Between Socioeconomic and Preterm Birth Factors With Educational Outcomes for Children Born 2008 or 2009 to 2011 or 2012 in England eTable 7. Unadjusted and Adjusted GEE Regression Models for the Association Between Socioeconomic and Preterm Birth Factors With Educational Outcomes (KS1 Writing and Science) for Children Born 2008 or 2009 to 2011 or 2012 in England eTable 8. Sensitivity Analysis Substituting Child’s Language for Ethnicity: Fully Adjusted GEE Regression Models for the Association of Socioeconomic and Preterm Birth Factors With Educational Outcomes for Children Born 2008 or 2009 to 2011 or 2012 in England eFigure 1. Heat Map of Correlations Between Outcomes and Covariates eFigure 2. Participant Flow of Cohort Included in the Study Born Between 2008 or 2009 and 2011 or 2012 in England eFigure 3. Distribution of Children Who Did Not Meet Expected Levels for Each Outcome in the Cohort and the Whole National Population eFigure 4. Proportion of Children Who Did Not Meet the Expected Level in Outcomes by Income Deprivation Affecting Children Index (IDACI) at Age 5 Years, Stratified by Gestational Age Category (23-26 Weeks and 27-31 Weeks) and the Overall Cohort eFigure 5. Proportion of Children Who Did Not Meet the Expected Level in Outcomes by Free School Meal Eligibility at Age 5 Years, Stratified by Gestational Age Category (23-26 Weeks and 27-31 Weeks) and the Overall Cohort eFigure 6. Forest Plot Showin [file jamanetwopen-e2623068-s001.pdf]

Supplemental Online Content

Haider S, Tsanas A, Batty GD, et al. Health and socioeconomic factors of preterm birth and school readiness and educational attainment. *JAMA Netw Open*. 2026;9(7):e2623068. doi:10.1001/jamanetworkopen/2026.23068

eMethods

eTable 1. Description of Variables

eTable 2. Distribution of Missing Data in Covariates

eTable 3. Characteristics of Children With and Without Sufficient Personal Identifiers for Linkage

eTable 4. Distribution of Children in the Preterm Cohort Who Did or Did Not Meet Expected Levels in Each Outcome by Birth Year

eTable 5. Distribution of Children in the Preterm Cohort Who Did Not Meet Expected Levels in Each Outcome by Gestational Age (Birth Years 2008-2009 to 2011-2012)

eTable 6. Unadjusted and Adjusted GEE Regression Models for the Association Between Socioeconomic and Preterm Birth Factors With Educational Outcomes for Children Born 2008 or 2009 to 2011 or 2012 in England

eTable 7. Unadjusted and Adjusted GEE Regression Models for the Association Between Socioeconomic and Preterm Birth Factors With Educational Outcomes (KS1 Writing and Science) for Children Born 2008 or 2009 to 2011 or 2012 in England

eTable 8. Sensitivity Analysis Substituting Child's Language for Ethnicity: Fully Adjusted GEE Regression Models for the Association of Socioeconomic and Preterm Birth Factors With Educational Outcomes for Children Born 2008 or 2009 to 2011 or 2012 in England

eFigure 1. Heat Map of Correlations Between Outcomes and Covariates

eFigure 2. Participant Flow of Cohort Included in the Study Born Between 2008 or 2009 and 2011 or 2012 in England

eFigure 3. Distribution of Children Who Did Not Meet Expected Levels for Each Outcome in the Cohort and the Whole National Population

eFigure 4. Proportion of Children Who Did Not Meet the Expected Level in Outcomes by Income Deprivation Affecting Children Index (IDACI) at Age 5 Years, Stratified by Gestational Age Category (23-26 Weeks and 27-31 Weeks) and the Overall Cohort

eFigure 5. Proportion of Children Who Did Not Meet the Expected Level in Outcomes by Free School Meal Eligibility at Age 5 Years, Stratified by Gestational Age Category (23-26 Weeks and 27-31 Weeks) and the Overall Cohort

eFigure 6. Forest Plot Showing the Unadjusted and Adjusted Associations Between Socioeconomic and Preterm Birth Factors With Educational Outcomes (Not Meeting the Expected Level in KS1 Writing or KS1 Science)

eFigure 7. Estimated Probability of Not Meeting Expected Levels in Educational Outcomes Across Index of Multiple Deprivation (IMD) at Birth, Stratified by Gestational Age

eReferences

This supplemental material has been provided by the authors to give readers additional information about their work.

© 2026 Haider S et al. *JAMA Network Open*

eMethods

1. Description of the datasets

The National Neonatal Research Database (NNRD) comprises a standard data extract (the Neonatal Data Set, an NHS Information Standard; DAPB1595) from Electronic Patient Records (EPR) very preterm infants. It contains data on all neonatal admissions from 2007 to the present, and approximately 450 de-identified entry items including demographic, diagnostic, daily, episodic and outcome variables.[1] All units in England have contributed data since 2012, providing whole population coverage for all preterm and sick infants admitted to neonatal units.[2]

The National Pupil Database (NPD) is a longitudinal administrative dataset collected and maintained by the Department for Education (DfE) of all children who attend state-funded schools in England.[3] This study used data on pupil characteristics, collected via the Pupil Level School Census three times per academic year (Autumn, Spring, and Summer terms), and educational attainment records. It includes the Early Years Foundation Stage Profile (EYFSP) at age 5 years, which is a summative assessment of a child's development that is used to support successful transition to year 1 of schooling and serves as a measure of school readiness. The NPD also includes a phonics check at age 6 years, and tests of reading, writing, mathematics, and science at age 7 years. All assessments are teacher-led.

Child-level linkage was performed by NHS Digital using personal identifiers (NHS numbers, date of birth, first name, surname, and postcodes) with subsequent deidentification, including removing name, date of birth, and exact date of neonatal discharge prior to study analysis.[4] Linkage to data from the Office for National Statistics provided information on registered deaths.

2. Missing data and imputation

Missingness was present for mode of delivery (8.5%), nutrition at discharge (6.5%), antenatal steroids (5.9%), and IMD (2.7%), Table S2. Prior to regression analyses, missing data on covariates were imputed using multiple imputation by chained equations, generating ten complete datasets under the assumption of missing at random. The imputation model incorporated all variables subsequently used in the regression analyses to preserve the multivariate relationships among covariates. Predictive mean matching or linear regression imputation was used for continuous variables, and logistic or multinomial regression for binary or categorial variables. Each imputed dataset was modelled independently, and results were pooled using Rubin's rule.

3. Predictive performance of the fully adjusted models

To evaluate out of sample predictive performance, the data were partitioned into training (70%) and test (30%) sets, stratifying by mother's ID to preserve clustering and prevent data leakage. The training set was multiply imputed (10 imputations), and GEE models were fitted on each complete dataset. Missing values in the test set were imputed using the median for continuous variables and the mode for categorical variables from the corresponding training set. Each trained model generated predictions on its paired test set and predicted probabilities for each participant were pooled by averaging across test sets. Performance was evaluated using AUC, sensitivity, specificity and accuracy.

© 2026 Haider S et al. *JAMA Network Open*

4. Extracted codes from NNRD to derive variables

Sepsis

"An infant fulfilled criteria for sepsis if they had either a positive blood culture report of the growth of any organism from the National Neonatal Audit Programme (NNAP) late onset infection list of "Clearly pathogenic organisms" OR "other organisms" which includes coagulase negative Staphylococcus.[5] OR a discharge diagnosis indicating one or more "clearly" or "other" pathogenic organisms from the NNAP lists.

Discharge diagnoses that match NNAP list of "pure pathogens" AND "other organisms" in the NNRD field "Principal diagnosis at discharge" are:

sepsis - confirmed bacterial (gram positive)
sepsis / septicaemia - confirmed with +ve microbiology
e.coli sepsis / septicaemia
candida sepsis / septicaemia
group b streptococcal sepsis / septicaemia (gbs)
staphylococcal sepsis / septicaemia
staph. aureus sepsis / septicaemia
sepsis / septicaemia - specified - klebsiella sp.
sepsis / septicaemia - specified - enterobacter sp.
sepsis / septicaemia - specified - pseudomonas sp.
extended beta lactamase coliform infection/sepsis
listeria sepsis / septicaemia / disseminated
sepsis - confirmed bacterial (streptococci b positive)
sepsis - confirmed bacterial (streptococci positive)
streptococcal sepsis / septicaemia
salmonella sepsis
sepsis due to streptococcus
umbilical sepsis / septicaemia- group b streptococcus

© 2026 Haider S et al. *JAMA Network Open*

Infant of a diabetic mother

To identify infants of mothers who had pre-existing or gestational diabetes:

Any of the following in ‘Principal diagnosis at discharge’:

“infant of diabetic mother"
"gestational diabetes” AND/OR

Any of the following in ‘Problems medical mother’:
“diabetes” AND/OR

Any of the following in ‘Problems during pregnancy:
“gestational diabetes”

Hypertensive disorders of pregnancy (HDP)

To identify infants of mothers with HDP, maternal HDP diagnoses were extracted from antenatal, delivery and neonatal NNRD data items.[6]

Any of the following in ‘Problems medical mother’:
“chronic hypertension” AND/OR

Any of the following in ‘Problems during pregnancy’:
“pregnancy-induced hypertension”
“pre-eclampsia”
“maternal HELLP” AND/OR

Any of the following in ‘Principal diagnosis at discharge’:
“mild pre-eclampsia”
“moderate pre-eclampsia”
“severe pre-eclampsia” AND/OR

Any of the following in ‘Drugs in labour’:
“antihypertensive”

© 2026 Haider S et al. *JAMA Network Open*

eTable 1. Description of Variables

Variable	Definition	Scale	Coding
School outcomes at age 5-7 years			
Early years Foundation Stage Profile (EYFSP)	Assessment at the end of Reception (age 5). Achieving at least the expected level in the Early Learning Goals related to communication and language, physical development, personal, social, and emotional development, literacy, and mathematics.	Binary	0=achieved GLD, 1= non-GLD
Phonics	Assessment at the end of Year 1 (age 6). Pupils scoring >32 on the 40-word test are considered to have met the expected standard. Pupils who do not meet the expected standard retake the check again at the end of year 2.	Binary	0= met the expected level, 1=did not meet the expected level
Key Stage 1 (KS1)	Assessment at the end of Year 2 (age 7) in reading, writing, mathematics, science. A child is assessed as meeting the expected level if they achieve level 2 or above.	Binary	0= at/above expected level, 1= did not meet the expected level (per domain)
Child's characteristics			
Academic year of birth	Defined to align with the academic calendar year (1st September to 31st August inclusive the following year) to ensure that the cohort's attainment outcomes corresponded to the relevant academic year for the educational assessments.	Categorical	0=2008/2009, 1=2009/2010, 2=2010/2011, 3=2011/2012
Season of birth	Birth month is divided into three terms based on the academic year: Summer-born is defined as births during May-August inclusive; Spring-born as births during January-April; and Autumn-born as births during September-December		0=Summer-born; 1=Autumn-born; 2=Spring-born
Gestational age	Age at birth in completed weeks.	Continuous	23-31 weeks inclusive
Gestational age group	Derived from the categorisation of the number of completed weeks of gestation at birth	Categorical	0=27-31 weeks, 1=23-26 weeks
Birth-weight z-score	Weight z-scores were calculated using the LMS method of Cole and Green using sex, gestational age, postnatal age and birth weight.[16-18]	Continuous	
Multiple gestation	Derived from the number of fetuses at birth	Binary	0=singleton, 1=multiple
Sex	Biological sex assigned at birth	Binary	1=male, 0=female,
Small for gestational age (SGA)	<10th centile for birth weight	Binary	
Mode of delivery	Mother's mode of delivery	Binary	0=C-section, 1=vaginal birth
Maternal characteristics			
Maternal age (years)	Age of mother at infant's birth	Continuous	
Smoking in pregnancy	Mother-confirmed smoking status during pregnancy	Binary	0=not smoking, 1=smoking
Hypertensive disorders of pregnancy	See extracted codes in Supplement	Binary	
Infant of a diabetic mother	See extracted codes in Supplement	Binary	

© 2026 Haider S et al. *JAMA Network Open*

Socio-economic/socio-demographic	`		
IMD decile	Index of Multiple Deprivation score: the decile measuring the deprivation score for the mother's location of residence at the time of the infant's birth.	Continuous	1-10. 1 being the least deprived and 10 being the most deprived
IDACI decile	Income Deprivation Affecting Children Index: the decile measuring the income deprivation score for the child's location of residence in the year of entry to Reception.	Continuous	1-10. 1 being the least deprived and 10 being the most deprived
Free school meal (FSM) eligibility	Child's FSM eligibility in the year of entry to Reception.	Binary	0=not eligible, 1=eligible
Child's language	Child's major language group. Derived as the mode of entries across all Pupil Census records.	Binary	0=English, 1=Not English
Maternal ethnicity	Ethnicities were self-reported and grouped according to the 2011 National Census categories[15]	Nominal	0=White, 1=Asian, 2=Black, 3=Mixed, 4=Other
Child's ethnicity	Derived as the mode of entries across all Pupil Census records.	Nominal	0=White, 1=Asian, 2=Black, 3=Chinese, 4=Mixed, 5=Other
Neonatal comorbidities			
Severe necrotising enterocolitis (NEC)	In accordance with the definition developed by Battersby et al.,[7] severe NEC is defined as NEC that required surgery or was confirmed at death. See extracted codes in Supplement.	Binary	0=No severe NEC detected, 1 =severe NEC present
Treated retinopathy of prematurity (ROP)	Whether a baby was surgically treated or given drugs (Avastin) for ROP	Binary	0 = No treatment given, 1 = treatment given
Sepsis	See extracted codes in Supplement	Binary	0 =No sepsis detected, 1= Sepsis detected
Chronic Lung disease/bronchopulmonary dysplasia	Respiratory support at 36 weeks corrected gestational age		0 – No respiratory support given at 36 weeks, 1 – Respiratory support at 36 weeks
Neonatal brain injuries			
Cystic periventricular leukomalacia (CPVL)	CPVL on cerebral ultrasound scan (CUSS) &/or in final diagnosis record	Binary	0=No CPVL of no record of CPVL, 1=CPVL detected
Porencephalic cyst	Porencephalic cyst visible on either the left or right hemispheres on CUSS &/or in final diagnosis record	Binary	0=No porencephalic cyst of no record of porencephalic cyst, 1= porencephalic cyst detected
Hydrocephalus	Post-haemorrhagic hydrocephalus visible on CUSS &/or in final diagnosis record	Binary	0=No hydrocephalus of no record of porencephalic

© 2026 Haider S et al. *JAMA Network Open*

			cyst, 1= hydrocephalus detected
Intraventricular haemorrhage	The most severe grade of IVH seen on left/right hemisphere on the CUS scan &/or in final diagnosis record	Categorical	0= no IVH, 1=IVH grade 1 or 2, 2=IVH grade 3 or 4
Interventions			
Ante-natal corticosteroids	Whether a complete course of antenatal steroid courses given during pregnancy	Binary	0=None or incomplete course, 1=complete course
Post-natal corticosteroids	Post-natal steroids received by 36 weeks' post-menstrual age	Binary	0=No, 1=yes
Enteral nutrition at discharge from neo-natal unit			
Type of milk	Discharged on breast milk versus, formula, mixed, or other feeding.	Binary	0=Breast milk, 1=Formula, 2=Mixed, 3=Other

© 2026 Haider S et al. *JAMA Network Open*

eTable 2. Distribution of Missing Data in Covariates

N=14,129

Variable	Frequency	% missing
Vaginal birth	1201	8.5
Nutrition at discharge	917	6.5
Antenatal corticosteroids	838	5.9
IMD at birth	386	2.7
Postnatal corticosteroids	305	2.2
Maternal age	45	0.3
Major language	15	0.1
Birthweight z-score	<10	<0.1
Gestational age	0	0
Free school meal (5 years)	0	0
Necrotising enterocolitis	0	0
Bronchopulmonary dysplasia	0	0
Sepsis	0	0
Diabetic mother	0	0
Hypertensive disorder of pregnancy	0	0
Retinopathy of prematurity	0	0
Maternal smoking	0	0
Cystic periventricular leukomalacia	0	0
Intraventricular haemorrhage	0	0
Porencephalic cyst	0	0
Hydrocephalus	0	0
Sex	0	0
Multiple birth	0	0
Term of birth	0	0

© 2026 Haider S et al. *JAMA Network Open*

eTable 3. Characteristics of Children With and Without Sufficient Personal Identifiers for Linkage

Frequencies (%) and median (IQR) are presented for categorical and continuous variables, respectively.

	**Insufficient personal identifiers for linkage**	**Identified for linkage**	**Total**
N	**6,617 (27.7%)**	**17,245 (72.3%)**	**23,862 (100%)**
Mother's age at birth (years)	30 (25 to 35)	30 (25 to 35)	30 (25 to 35)
Birth-weight z-score	-0.28 (-0.87 to 0.27)	-0.22 (-0.82 to 0.31)	-0.23 (-0.83 to 0.30)
Gestational age (completed weeks)			
23	124 (1.9%)	176 (1.0%)	300 (1.3%)
24	344 (5.2%)	584 (3.4%)	928 (3.9%)
25	396 (6.0%)	834 (4.8%)	1,230 (5.2%)
26	543 (8.2%)	1,328 (7.7%)	1,871 (7.8%)
27	713 (10.8%)	1,698 (9.8%)	2,411 (10.1%)
28	881 (13.3%)	2,255 (13.1%)	3,136 (13.1%)
29	917 (13.9%)	2,588 (15.0%)	3,505 (14.7%)
30	1,140 (17.2%)	3,321 (19.3%)	4,461 (18.7%)
31	1,559 (23.6%)	4,461 (25.9%)	6,020 (25.2%)
Small for gestational age	992 (15.0%)	2,351 (13.6%)	3,343 (14.0%)
Birth multiplicity			
Singleton	4,729 (71.5%)	12,547 (72.8%)	17,276 (72.4%)
Multiple	1,885 (28.5%)	4,693 (27.2%)	6,578 (27.6%)
Sex at birth			
Female	3,067 (46.4%)	8,010 (46.4%)	11,077 (46.4%)
Male	3,550 (53.6%)	9,235 (53.6%)	12,785 (53.6%)
IMD decile at birth			
1 Most deprived	974 (16.0%)	3,025 (18.1%)	3,999 (17.5%)
2	808 (13.3%)	2,462 (14.7%)	3,270 (14.3%)
3	819 (13.4%)	2,101 (12.6%)	2,920 (12.8%)
4	711 (11.7%)	1,739 (10.4%)	2,450 (10.7%)
5	568 (9.3%)	1,616 (9.7%)	2,184 (9.6%)
6	503 (8.3%)	1,360 (8.1%)	1,863 (8.2%)
7	458 (7.5%)	1,256 (7.5%)	1,714 (7.5%)
8	436 (7.2%)	1,152 (6.9%)	1,588 (7.0%)
9	414 (6.8%)	1,030 (6.2%)	1,444 (6.3%)
10 Least deprived	404 (6.6%)	984 (5.9%)	1,388 (6.1%)
Maternal ethnicity			
Asian	868 (14.0%)	1,806 (11.1%)	2,674 (11.9%)
Black	779 (12.5%)	1,497 (9.2%)	2,276 (10.1%)
Mixed	118 (1.9%)	263 (1.6%)	381 (1.7%)
Other	163 (2.6%)	312 (1.9%)	475 (2.1%)
White	4,280 (68.9%)	12,462 (76.3%)	16,742 (74.3%)
Maternal smoking at birth	1,129 (17.1%)	3,067 (17.8%)	4,196 (17.6%)
Complete course of ante-natal corticosteroids	3,924 (66.8%)	11,369 (70.1%)	15,293 (69.2%)
Post-natal corticosteroids	518 (8.1%)	1,229 (7.3%)	1,747 (7.5%)
Infant of a diabetic mother	195 (2.9%)	492 (2.9%)	687 (2.9%)
Hypertensive disorders of pregnancy	799 (12.1%)	2,199 (12.8%)	2,998 (12.6%)
Cystic periventricular leukomalacia	95 (1.4%)	277 (1.6%)	372 (1.6%)

	Insufficient personal identifiers for linkage	Identified for linkage	Total
intraventricular haemorrhage (IVH)			
No IVH	5,794 (87.6%)	14,954 (86.7%)	20,748 (86.9%)
IVH grade I-II	567 (8.6%)	1,788 (10.4%)	2,355 (9.9%)
IVH grade III-IV	256 (3.9%)	503 (2.9%)	759 (3.2%)
Porencephalic cyst	57 (0.9%)	229 (1.3%)	286 (1.2%)
Hydrocephalus	90 (1.4%)	184 (1.1%)	274 (1.1%)
Sepsis	328 (5.0%)	874 (5.1%)	1,202 (5.0%)
Necrotising enterocolitis	269 (4.1%)	418 (2.4%)	687 (2.9%)
Retinopathy of prematurity	204 (3.1%)	498 (2.9%)	702 (2.9%)
Bronchopulmonary dysplasia	1,864 (30.2%)	4,636 (27.5%)	6,500 (28.2%)
Enteral milk at discharge			
Breast	1,697 (29.1%)	4,776 (29.9%)	6,473 (29.7%)
Formula	2,173 (37.2%)	6,213 (39.0%)	8,386 (38.5%)
Other	593 (10.2%)	1,594 (10.0%)	2,187 (10.0%)
Mixed	1,376 (23.6%)	3,364 (21.1%)	4,740 (21.8%)

© 2026 Haider S et al. *JAMA Network Open*

eTable 4. Distribution of Children in the Preterm Cohort Who Did or Did Not Meet Expected Levels in Each Outcome by Birth Year

	EYFSP (5 years)			Phonics (6 years)			KS1: Reading (7 years)			KS1: Writing (7 years)			KS1: Maths (7 years)			KS1: Science (7 years)		
**Birth year**	**Met expected level**	**Did not meet expected level**	**TOTAL**	**Met expected level**	**Did not meet expected level**	**TOTAL**	**Met expected level**	**Did not meet expected level**	**TOTAL**	**Met expected level**	**Did not meet expected level**	**TOTAL**	**Met expected level**	**Did not meet expected level**	**TOTAL**	**Met expected level**	**Did not meet expected level**	**TOTAL**
**2008/2009**	710	1,173	1,883	1,499	380	1,879	1,080	770	1,850	857	993	1,850	920	930	1,850	1,187	663	1,850
	37.7	62.3	100	79.8	20.2	100	58.4	41.6	100	46.3	53.7	100	49.7	50.3	100	64.2	35.8	100
**2009/2010**	1,615	2,247	3,862	3,032	802	3,834	2,199	1,600	3,799	1,798	2,001	3,799	1,942	1,857	3,799	2,427	1,372	3,799
	41.8	58.2	100	79.1	20.9	100	57.9	42.1	100	47.3	52.7	100	51.1	48.9	100	63.9	36.1	100
**2010/2011**	2,209	2,643	4,852	3,863	992	4,855	2,813	1,968	4,781	2,379	2,402	4,781	2,548	2,233	4,781	3,090	1,691	4,781
	45.5	54.5	100	79.6	20.4	100	58.8	41.2	100	49.8	50.2	100	53.3	46.7	100	64.6	35.4	100
**2011/2012**	2,392	2,539	4,931	3,884	1,052	4,936	2,695	2,016	4,711	2,318	2,393	4,711	2,514	2,196	4,710	2,988	1,723	4,711
	48.5	51.5	100	78.7	21.3	100	57.2	42.8	100	49.2	50.8	100	53.4	46.6	100	63.4	36.6	100
**Total**	6,926	8,602	15,528	12,278	3,226	15,504	8,787	6,354	15,141	7,352	7,789	15,141	7,924	7,216	15,140	9,692	5,449	15,141
**4-year mean**	43.4	56.6	100	79.3	20.7	100	58.1	41.9	100	48.2	51.8	100	51.9	48.1	100	64.0	36.0	100

© 2026 Haider S et al. *JAMA Network Open*

eTable 5. Distribution of Children in the Preterm Cohort Who Did Not Meet Expected Levels in Each Outcome by Gestational Age (Birth Years 2008-2009 to 2011-2012)

	EYFSP (5 years)		Phonics (6 years)		KS1 Reading (7 years)		KS1 Writing (7 years)		KS1 Maths (7 years)		KS1 Science (7 years)	
Gestation (weeks)	n/N	%	n/N	%	n/N	%	n/N	%	n/N	%	n/N	%
23	120/136	88.2	70/138	50.7	96/131	73.3	113/131	86.3	111/131	84.7	97/131	74
24	377/484	77.9	221/496	44.6	321/479	67	363/479	75.8	369/479	77	306/479	63.9
25	503/711	70.7	244/725	33.7	395/695	56.8	455/695	65.5	452/695	65	386/695	55.5
26	737/1170	63	296/1166	25.4	531/1132	46.9	657/1132	58	641/1132	56.6	491/1132	43.4
27	908/1506	60.3	335/1508	22.2	672/1463	45.9	804/1463	55	771/1463	52.7	595/1463	40.7
28	1206/2058	58.6	456/2047	22.3	864/1998	43.2	1063/1998	53.2	993/1998	49.7	761/1998	38.1
29	1242/2337	53.1	424/2333	18.2	912/2289	39.8	1142/2289	49.9	1038/2289	45.3	745/2289	32.5
30	1517/3043	49.9	542/3022	17.9	1091/2958	36.9	1364/2958	46.1	1240/2958	41.9	911/2958	30.8
31	1992/4083	48.8	638/4069	15.7	1472/3996	36.8	1828/3996	45.7	1601/3995	40.1	1157/3996	29

© 2026 Haider S et al. *JAMA Network Open*

eTable 6. Unadjusted and Adjusted GEE Regression Models for the Association Between Socioeconomic and Preterm Birth Factors With Educational Outcomes for Children Born 2008 or 2009 to 2011 or 2012 in England

Results are expressed as odds ratios with 95% confidence intervals. The adjusted model includes all covariates listed for unadjusted models (N=14,129).

Covariate		EYFSP (age 5)		Phonics (age 6)		KS1_Reading (age 7)		KS1_Maths (age 7)	
	Freq.	Unadjusted	Adjusted	Unadjusted	Adjusted	Unadjusted	Adjusted	Unadjusted	Adjusted
		OR/ 95% CI	OR/ 95% CI	OR/ 95% CI	OR/ 95% CI	OR/ 95% CI	OR/ 95% CI	OR/ 95% CI	OR/ 95% CI
**Gestational age (completed weeks)**									
23-24	544	4.42	2.86	4.62	1.91	3.59	2.15	5.3	2.93
		[3.52,5.55]	[2.19,3.73]	[3.80,5.61]	[1.48,2.48]	[2.96,4.37]	[1.69,2.72]	[4.27,6.57]	[2.28,3.77]
25	634	2.59	1.89	2.78	1.37	2.14	1.42	2.65	1.67
		[2.15,3.13]	[1.52,2.35]	[2.29,3.38]	[1.09,1.73]	[1.80,2.54]	[1.16,1.74]	[2.22,3.16]	[1.36,2.05]
26	1048	1.77	1.34	1.9	1.09	1.52	1.1	1.92	1.35
		[1.53,2.05]	[1.14,1.59]	[1.59,2.25]	[0.89,1.33]	[1.32,1.75]	[0.93,1.30]	[1.67,2.22]	[1.15,1.59]
27	1363	1.58	1.25	1.55	1.01	1.45	1.11	1.69	1.27
		[1.39,1.80]	[1.08,1.45]	[1.31,1.82]	[0.84,1.21]	[1.27,1.65]	[0.96,1.28]	[1.49,1.92]	[1.10,1.46]
28	1872	1.44	1.24	1.57	1.18	1.31	1.09	1.5	1.22
		[1.28,1.62]	[1.09,1.41]	[1.35,1.82]	[1.00,1.38]	[1.16,1.47]	[0.96,1.23]	[1.33,1.68]	[1.08,1.38]
29	2158	1.21	1.12	1.24	1.06	1.16	1.06	1.28	1.17
		[1.08,1.35]	[1.00,1.26]	[1.07,1.44]	[0.91,1.24]	[1.04,1.30]	[0.94,1.20]	[1.15,1.43]	[1.04,1.32]
30	2789	1.06	1.02	1.18	1.11	1.01	0.97	1.09	1.06
		[0.96,1.17]	[0.92,1.14]	[1.03,1.35]	[0.96,1.28]	[0.91,1.12]	[0.87,1.09]	[0.99,1.21]	[0.95,1.18]
31: reference	3721	1	1	1	1	1	1	1	1

© 2026 Haider S et al. *JAMA Network Open*

Covariate	Freq.	EYFSP (age 5)		Phonics (age 6)		KS1_Reading (age 7)		KS1_Maths (age 7)	
		Unadjusted	Adjusted	Unadjusted	Adjusted	Unadjusted	Adjusted	Unadjusted	Adjusted
**Child characteristics**									
Birthweight z-score	14,100	0.87	0.9	0.85	0.87	0.88	0.9	0.83	0.85
		[0.84,0.90]	[0.86,0.94]	[0.81,0.89]	[0.82,0.92]	[0.85,0.91]	[0.86,0.94]	[0.80,0.86]	[0.81,0.88]
Term of birth - Autumn: reference	4382	1	1	1	1	1	1	1	1
Term of birth - Spring	4797	1.52	1.64	1.2	1.24	1.27	1.32	1.29	1.35
		[1.39,1.65]	[1.50,1.80]	[1.08,1.34]	[1.10,1.39]	[1.16,1.39]	[1.21,1.45]	[1.18,1.41]	[1.23,1.47]
Term of birth - Summer	4950	2.31	2.64	1.44	1.55	1.63	1.74	1.81	1.97
		[2.11,2.51]	[2.41,2.90]	[1.29,1.60]	[1.38,1.73]	[1.49,1.77]	[1.59,1.91]	[1.66,1.97]	[1.81,2.16]
Singleton birth (vs. multiple birth)	10,845	0.85	0.93	0.94	1	0.98	1.07	0.91	0.96
		[0.78,0.93]	[0.85,1.03]	[0.84,1.05]	[0.89,1.13]	[0.89,1.07]	[0.97,1.17]	[0.83,1.00]	[0.88,1.06]
Male (vs. female)	7,493	1.76	1.96	1.44	1.5	1.37	1.44	1.05	1.07
		[1.64,1.88]	[1.82,2.11]	[1.33,1.57]	[1.37,1.64]	[1.28,1.47]	[1.34,1.55]	[0.98,1.12]	[1.00,1.15]
Vaginal birth (vs c-section)	7,540	0.91	1.07	0.86	1.01	0.95	1.1	0.92	1.04
		[0.85,0.98]	[0.98,1.17]	[0.79,0.94]	[0.91,1.13]	[0.88,1.02]	[1.00,1.20]	[0.86,0.99]	[0.95,1.13]

© 2026 Haider S et al. *JAMA Network Open*

Covariate	Freq.	EYFSP (age 5) Unadjusted	EYFSP (age 5) Adjusted	Phonics (age 6) Unadjusted	Phonics (age 6) Adjusted	KS1_Reading (age 7) Unadjusted	KS1_Reading (age 7) Adjusted	KS1_Maths (age 7) Unadjusted	KS1_Maths (age 7) Adjusted
Major language at home not English (vs. English)	2,164	1.21	1.3	0.87	0.93	0.97	1.04	0.94	0.98
		[1.10,1.33]	[1.17,1.45]	[0.77,0.98]	[0.81,1.07]	[0.88,1.06]	[0.94,1.16]	[0.86,1.03]	[0.88,1.08]
**Socio-economic status**									
IMD quintile at birth 1 (most deprived)	4,585	1.85	1.27	1.95	1.39	1.87	1.32	1.65	1.17
		[1.64,2.08]	[1.11,1.45]	[1.66,2.30]	[1.16,1.66]	[1.65,2.12]	[1.16,1.52]	[1.46,1.86]	[1.02,1.33]
IMD quintile 2	3,207	1.46	1.15	1.77	1.44	1.50	1.2	1.37	1.08
		[1.29,1.66]	[1.01,1.32]	[1.49,2.10]	[1.20,1.73]	[1.32,1.72]	[1.05,1.39]	[1.20,1.55]	[0.94,1.24]
IMD quintile 3	2,459	1.2	1.04	1.55	1.4	1.32	1.17	1.23	1.08
		[1.05,1.37]	[0.91,1.20]	[1.29,1.86]	[1.16,1.69]	[1.15,1.52]	[1.01,1.35]	[1.07,1.40]	[0.94,1.25]
IMD quintile 4	1,905	1.06	0.98	1.23	1.17	1.12	1.04	1.05	0.98
		[0.92,1.22]	[0.85,1.13]	[1.01,1.50]	[0.95,1.43]	[0.96,1.30]	[0.90,1.21]	[0.91,1.21]	[0.84,1.14]
IMD quintile 5 (least deprived): reference	1,587	1	1	1	1	1	1	1	1
Free school meal eligible at 5 years	3,354	2.21	1.89	2.18	1.85	2.13	1.74	2	1.68
		[2.02,2.40]	[1.72,2.08]	[1.98,2.38]	[1.67,2.06]	[1.97,2.31]	[1.59,1.90]	[1.85,2.17]	[1.53,1.84]

© 2026 Haider S et al. *JAMA Network Open*

Covariate	Freq.	EYFSP (age 5)		Phonics (age 6)		KS1_Reading (age 7)		KS1_Maths (age 7)	
		Unadjusted	Adjusted	Unadjusted	Adjusted	Unadjusted	Adjusted	Unadjusted	Adjusted
**Maternal**									
		[1.10,1.33]	[1.17,1.45]	[0.77,0.98]	[0.81,1.07]	[0.88,1.06]	[0.94,1.16]	[0.86,1.03]	[0.88,1.08]
Maternal smoking at birth	2,660	1.68	1.28	1.69	1.22	1.77	1.31	1.61	1.19
		[1.53,1.84]	[1.15,1.42]	[1.52,1.86]	[1.08,1.37]	[1.62,1.93]	[1.19,1.44]	[1.48,1.76]	[1.08,1.31]
Mother's age at birth (years)	14,084	0.97	0.99	0.97	1	0.97	0.98	0.97	0.99
		[0.97,0.98]	[0.98,0.99]	[0.97,0.98]	[0.99,1.00]	[0.96,0.97]	[0.98,0.99]	[0.96,0.97]	[0.98,0.99]
Infant of a diabetic mother	2,660	1.09	1.26	0.88	1.09	1.05	1.26	1.07	1.36
		[0.89,1.33]	[1.01,1.57]	[0.68,1.15]	[0.83,1.44]	[0.85,1.29]	[1.02,1.57]	[0.87,1.31]	[1.10,1.69]
Hypertensive disorder of pregnancy	1,886	0.87	0.94	0.81	0.91	0.84	0.91	0.92	0.94
		[0.79,0.96]	[0.83,1.05]	[0.71,0.93]	[0.79,1.06]	[0.76,0.93]	[0.81,1.02]	[0.83,1.01]	[0.84,1.05]
**Interventions**									
Complete course of ante-natal corticosteroids	9,285	0.92	0.94	0.77	0.8	0.88	0.9	0.89	0.9
		[0.85,1.00]	[0.87,1.03]	[0.71,0.85]	[0.72,0.88]	[0.82,0.95]	[0.83,0.97]	[0.83,0.96]	[0.83,0.97]
Post-natal corticosteroids	937	2.68	1.37	3.21	1.58	2.42	1.32	2.68	1.28
		[2.30,3.13]	[1.13,1.65]	[2.80,3.68]	[1.32,1.87]	[2.11,2.77]	[1.12,1.56]	[2.33,3.09]	[1.08,1.52]

© 2026 Haider S et al. *JAMA Network Open*

Covariate		EYFSP (age 5)		Phonics (age 6)		KS1_Reading (age 7)		KS1_Maths (age 7)	
	**Freq.**	**Unadjusted**	**Adjusted**	**Unadjusted**	**Adjusted**	**Unadjusted**	**Adjusted**	**Unadjusted**	**Adjusted**
**Neonatal brain injuries**									
Cystic periventricular leukomalacia	216	3.77	3.05	4.88	3.58	2.96	2.26	3.42	2.49
		[2.64,5.37]	[2.04,4.58]	[3.72,6.39]	[2.57,4.99]	[2.22,3.95]	[1.62,3.14]	[2.52,4.65]	[1.74,3.56]
No intra-ventricular haemorrhage (IVH): reference	12,286								
		1	1	1	1	1	1	1	1
IVH grade I-II	1,473	1.24	0.95	1.44	1.07	1.2	0.94	1.4	1.08
		[1.11,1.39]	[0.84,1.07]	[1.26,1.63]	[0.93,1.23]	[1.07,1.33]	[0.84,1.06]	[1.26,1.56]	[0.96,1.21]
IVH grade III-IV	370	3.56	1.94	4.5	2.04	3.07	1.73	3.46	1.75
		[2.76,4.59]	[1.44,2.61]	[3.66,5.54]	[1.55,2.68]	[2.48,3.81]	[1.34,2.25]	[2.74,4.38]	[1.32,2.31]
Porencephalic cysts	179	2.66	1.31	3.11	1.06	2.65	1.38	3.32	1.62
		[1.90,3.71]	[0.88,1.94]	[2.31,4.19]	[0.71,1.58]	[1.95,3.61]	[0.96,1.99]	[2.37,4.64]	[1.10,2.37]
Hydrocephalus	145	4.41	2.47	6.18	2.9	3.58	1.95	5.15	2.82
		[2.85,6.81]	[1.48,4.13]	[4.37,8.75]	[1.89,4.46]	[2.50,5.14]	[1.28,2.96]	[3.35,7.90]	[1.71,4.64]
**Neonatal morbidities**									
Sepsis	688	1.48	1.07	1.75	1.15	1.55	1.15	1.6	1.11
		[1.27,1.73]	[0.90,1.27]	[1.48,2.06]	[0.96,1.38]	[1.33,1.80]	[0.98,1.35]	[1.37,1.87]	[0.94,1.31]

Covariate		EYFSP (age 5)		Phonics (age 6)		KS1_Reading (age 7)		KS1_Maths (age 7)	
	**Freq.**	**Unadjusted**	**Adjusted**	**Unadjusted**	**Adjusted**	**Unadjusted**	**Adjusted**	**Unadjusted**	**Adjusted**
Necrotising enterocolitis	302	2.52	1.6	3.03	1.85	2.49	1.7	2.91	1.8
		[1.94,3.29]	[1.20,2.14]	[2.41,3.80]	[1.43,2.40]	[1.96,3.15]	[1.32,2.17]	[2.26,3.74]	[1.37,2.35]
Retinopathy of prematurity	383	3.32	1.46	3.62	1.5	2.91	1.39	3.53	1.48
		[2.58,4.26]	[1.10,1.93]	[2.95,4.45]	[1.17,1.93]	[2.34,3.62]	[1.09,1.77]	[2.79,4.48]	[1.13,1.92]
Bronchopulmonary dysplasia	3,663	1.95	1.3	2.28	1.47	1.87	1.31	2.1	1.35
		[1.80,2.12]	[1.18,1.44]	[2.09,2.49]	[1.30,1.65]	[1.73,2.02]	[1.19,1.45]	[1.95,2.27]	[1.22,1.49]
**Nutrition at discharge**									
Breast milk: reference	3,816	1	1	1	1	1	1	1	1
Formula milk	5,355	1.9	1.51	2.22	1.66	2.08	1.57	2.06	1.59
		[1.74,2.07]	[1.37,1.66]	[1.97,2.50]	[1.46,1.88]	[1.90,2.28]	[1.42,1.73]	[1.88,2.25]	[1.45,1.76]
Other milk	1,334	2.35	1.64	3.05	1.87	2.45	1.62	2.69	1.77
		[2.07,2.67]	[1.42,1.89]	[2.62,3.55]	[1.58,2.22]	[2.17,2.78]	[1.41,1.86]	[2.37,3.06]	[1.54,2.03]
Mixed milk	2,707	1.37	1.19	1.5	1.29	1.41	1.22	1.38	1.18
		[1.24,1.52]	[1.07,1.33]	[1.30,1.73]	[1.11,1.49]	[1.27,1.56]	[1.09,1.35]	[1.24,1.52]	[1.06,1.32]
N			14,129		14,129		14,129		14,129
**Out of sample predictive performance**									

© 2026 Haider S et al. *JAMA Network Open*

Covariate		EYFSP (age 5)		Phonics (age 6)		KS1_Reading (age 7)		KS1_Maths (age 7)	
	Freq.	Unadjusted	Adjusted	Unadjusted	Adjusted	Unadjusted	Adjusted	Unadjusted	Adjusted
AUC			0.707		0.7		0.681		0.696
Sensitivity (positive class=failed to meet expected level)			0.545		0.61		0.54		0.528
Specificity			0.757		0.702		0.726		0.76
Accuracy			0.639		0.684		0.638		0.678
Balanced accuracy			0.651		0.656		0.633		0.644

© 2026 Haider S et al. *JAMA Network Open*

eTable 7. Unadjusted and Adjusted GEE Regression Models for the Association Between Socioeconomic and Preterm Birth Factors With Educational Outcomes (KS1 Writing and Science) for Children Born 2008 or 2009 to 2011 or 2012 in England

Results are expressed at odds ratios with 95% confidence intervals. The adjusted model includes all covariates listed for unadjusted models. N=14,129.

		KS1_Writing		KS1_Science	
**Covariate**	**Freq.**	**Unadjusted**	**Adjusted**	**Unadjusted**	**Adjusted**
		**OR/ 95% CI**	**OR/ 95% CI**	**OR/ 95% CI**	**OR/ 95% CI**
**Gestational age (completed weeks)**					
23-24	544	3.99	2.59	4.53	2.62
		[3.23,4.93]	[2.01,3.34]	[3.74,5.50]	[2.07,3.33]
25	634	2.18	1.56	2.93	1.9
		[1.82,2.60]	[1.27,1.91]	[2.46,3.49]	[1.55,2.34]
26	1048	1.63	1.23	1.87	1.32
		[1.41,1.88]	[1.04,1.45]	[1.62,2.17]	[1.11,1.56]
27	1363	1.44	1.14	1.68	1.26
		[1.26,1.64]	[0.99,1.31]	[1.47,1.93]	[1.08,1.46]
28	1872	1.34	1.14	1.51	1.23
		[1.19,1.50]	[1.01,1.29]	[1.34,1.71]	[1.08,1.40]
29	2158	1.21	1.13	1.21	1.09
		[1.08,1.35]	[1.00,1.27]	[1.08,1.37]	[0.96,1.24]
30	2789	1.015	0.98	1.101	1.06
		[0.92,1.12]	[0.88,1.09]	[0.98,1.23]	[0.95,1.20]
31: reference	3721	1	1	1	1
**Child characteristics**					
Birthweight z-score	14,100	0.88	0.89	0.84	0.86
		[0.85,0.91]	[0.85,0.93]	[0.80,0.87]	[0.82,0.90]
Term of birth - Autumn: reference	4382	1	1	1	1
Term of birth - Spring	4797	1.24	1.29	1.26	1.32
		[1.14,1.35]	[1.18,1.42]	[1.15,1.38]	[1.20,1.45]
Term of birth - Summer	4950	1.69	1.84	1.68	1.83
		[1.55,1.84]	[1.68,2.01]	[1.54,1.84]	[1.67,2.01]
Singleton birth (vs. multiple birth)	10,845	0.88	0.96	0.86	0.93
		[0.81,0.97]	[0.87,1.05]	[0.78,0.94]	[0.84,1.03]
Male (vs. female)	7,493	1.69	1.83	1.24	1.31
		[1.58,1.81]	[1.70,1.96]	[1.16,1.33]	[1.21,1.41]
Vaginal birth (vs c-section)	7,540	0.92	1.09	0.93	1.09
		[0.86,0.99]	[1.00,1.19]	[0.86,1.00]	[1.00,1.20]
Major language at home not English (vs. English)	2,164	0.9	0.96	1.11	1.16

© 2026 Haider S et al. *JAMA Network Open*

		KS1_Writing		KS1_Science	
Covariate	Freq.	Unadjusted	Adjusted	Unadjusted	Adjusted
		[0.82,0.99]	[0.86,1.06]	[1.01,1.22]	[1.04,1.29]
**Socio-economic status**					
IMD quintile at birth 1 (most deprived)	4,585	1.698	1.22	2.178	1.48
		[1.51,1.91]	[1.07,1.39]	[1.91,2.49]	[1.28,1.71]
IMD quintile 2	3,207	1.37	1.1	1.665	1.28
		[1.21,1.56]	[0.96,1.26]	[1.45,1.92]	[1.10,1.49]
IMD quintile 3	2,459	1.23	1.09	1.362	1.17
		[1.08,1.41]	[0.95,1.26]	[1.17,1.58]	[1.00,1.37]
IMD quintile 4	1,905	1.04	0.97	1.166	1.07
		[0.91,1.19]	[0.84,1.12]	[1.00,1.37]	[0.91,1.26]
IMD quintile at birth 5 (least deprived): reference	1,587	1	1	1	1
Free school meal eligible at 5 years	3,354	2.03	1.65	2.2	1.78
		[1.87,2.20]	[1.50,1.80]	[2.02,2.38]	[1.62,1.95]
**Maternal characteristics**					
Maternal smoking at birth	2,660	1.84	1.37	1.8	1.34
		[1.68,2.01]	[1.24,1.51]	[1.64,1.96]	[1.22,1.48]
Mother's age at birth (years)	14,084	0.97	0.98	0.97	0.99
		[0.96,0.97]	[0.98,0.99]	[0.96,0.97]	[0.98,0.99]
Infant of a diabetic mother	2,660	1.2	1.51	1.21	1.51
		[0.98,1.46]	[1.22,1.87]	[0.99,1.49]	[1.20,1.89]
Hypertensive disorder of pregnancy	1,886	0.82	0.88	0.88	0.92
		[0.75,0.91]	[0.79,0.99]	[0.80,0.98]	[0.82,1.04]
**Interventions**					
Complete course of ante-natal corticosteroids	9,285	0.87	0.88	0.86	0.86
		[0.80,0.93]	[0.82,0.96]	[0.79,0.93]	[0.79,0.94]
Post-natal corticosteroids	937	2.42	1.26	2.57	1.21
		[2.10,2.79]	[1.06,1.50]	[2.25,2.94]	[1.03,1.43]
**Neonatal brain injuries**					
Cystic periventricular leukomalacia	216	3.05	2.35	3.21	2.4
		[2.23,4.16]	[1.65,3.35]	[2.44,4.22]	[1.72,3.36]
No intra-ventricular haemorrhage (IVH): reference	12,286	1	1	1	1
IVH grade I-II	1,473	1.26	0.99	1.38	1.05
		[1.13,1.41]	[0.88,1.12]	[1.24,1.54]	[0.93,1.19]
IVH grade III-IV	370	2.88	1.48	3.14	1.53
		[2.29,3.62]	[1.13,1.94]	[2.55,3.86]	[1.17,1.99]
Porencephalic cysts	179	3.06	1.69	2.94	1.43

© 2026 Haider S et al. *JAMA Network Open*

		KS1_Writing		KS1_Science	
**Covariate**	**Freq.**	**Unadjusted**	**Adjusted**	**Unadjusted**	**Adjusted**
		[2.18,4.31]	[1.15,2.49]	[2.18,3.97]	[0.99,2.06]
Hydrocephalus	145	4.3	2.61	4.6	2.7
		[2.84,6.52]	[1.63,4.18]	[3.20,6.60]	[1.73,4.22]
**Neonatal morbidities**					
Sepsis	688	1.48	1.09	1.7	1.18
		[1.26,1.72]	[0.93,1.29]	[1.46,1.98]	[1.00,1.39]
Necrotising enterocolitis	302	2.74	1.84	3.06	1.96
		[2.12,3.55]	[1.40,2.41]	[2.42,3.86]	[1.53,2.52]
Retinopathy of prematurity	383	3.17	1.5	3.31	1.42
		[2.49,4.02]	[1.15,1.95]	[2.67,4.09]	[1.11,1.82]
Bronchopulmonary dysplasia	3,663	1.87	1.26	2.1	1.37
		[1.73,2.02]	[1.14,1.39]	[1.95,2.27]	[1.24,1.51]
**Nutrition at discharge**					
Breast milk: reference	3,816	1	1	1	1
Formula milk	5,355	1.97	1.5	2.16	1.59
		[1.80,2.15]	[1.36,1.65]	[1.97,2.37]	[1.43,1.77]
Other milk	1,334	2.4	1.62	2.75	1.71
		[2.11,2.73]	[1.41,1.87]	[2.42,3.13]	[1.48,1.97]
Mixed milk	2,707	1.29	1.12	1.51	1.27
		[1.17,1.43]	[1.01,1.25]	[1.35,1.68]	[1.14,1.43]
**Out of sample predictive performance**					
AUC			0.675		0.685
Sensitivity (positive class=failed to meet expected level)			0.633		0.631
Specificity			0.626		0.645
Accuracy			0.629		0.638
Balanced accuracy			0.630		0.638

© 2026 Haider S et al. *JAMA Network Open*

eTable 8. Sensitivity Analysis Substituting Child's Language for Ethnicity: Fully Adjusted GEE Regression Models for the Association of Socioeconomic and Preterm Birth Factors With Educational Outcomes for Children Born 2008 or 2009 to 2011 or 2012 in England

Model includes all other covariates reported in fully adjusted models in Table 2 and Table S4. N=14,129.

Results are expressed at odds ratios with 95% confidence intervals.

	Freq.	EYFSP OR/ 95% CI	Phonics OR/ 95% CI	KS1_Reading OR/ 95% CI	KS1_Writing OR/ 95% CI	KS1_Maths OR/ 95% CI	KS1_Science OR/ 95% CI
**Child's ethnicity**							
White: reference	10,421	1	1	1	1	1	1
Asian	1,336	1.24	0.92	0.98	0.87	0.88	1.15
		[1.08,1.41]	[0.78,1.09]	[0.86,1.11]	[0.77,0.99]	[0.78,1.01]	[1.00,1.31]
Black	1,064	1	0.89	0.89	0.84	0.99	1.09
		[0.86,1.16]	[0.74,1.07]	[0.77,1.03]	[0.73,0.97]	[0.86,1.14]	[0.94,1.27]
Mixed	984	0.81	0.8	0.88	0.92	0.98	0.93
		[0.70,0.94]	[0.67,0.96]	[0.75,1.02]	[0.79,1.06]	[0.84,1.13]	[0.80,1.09]
Other	186	1.4	0.71	0.67	0.69	0.6	0.9
		[1.01,1.94]	[0.44,1.14]	[0.48,0.94]	[0.50,0.94]	[0.43,0.84]	[0.64,1.27]

© 2026 Haider S et al. *JAMA Network Open*

eFigure 1. Heat Map of Correlations Between Outcomes and Covariates

Pearson's correlation coefficient was used to quantify the strength of the association between two continuous variables, the tetrachoric coefficient for two binary variables, and the point-biserial correlation coefficient for pairs of continuous and binary variables.

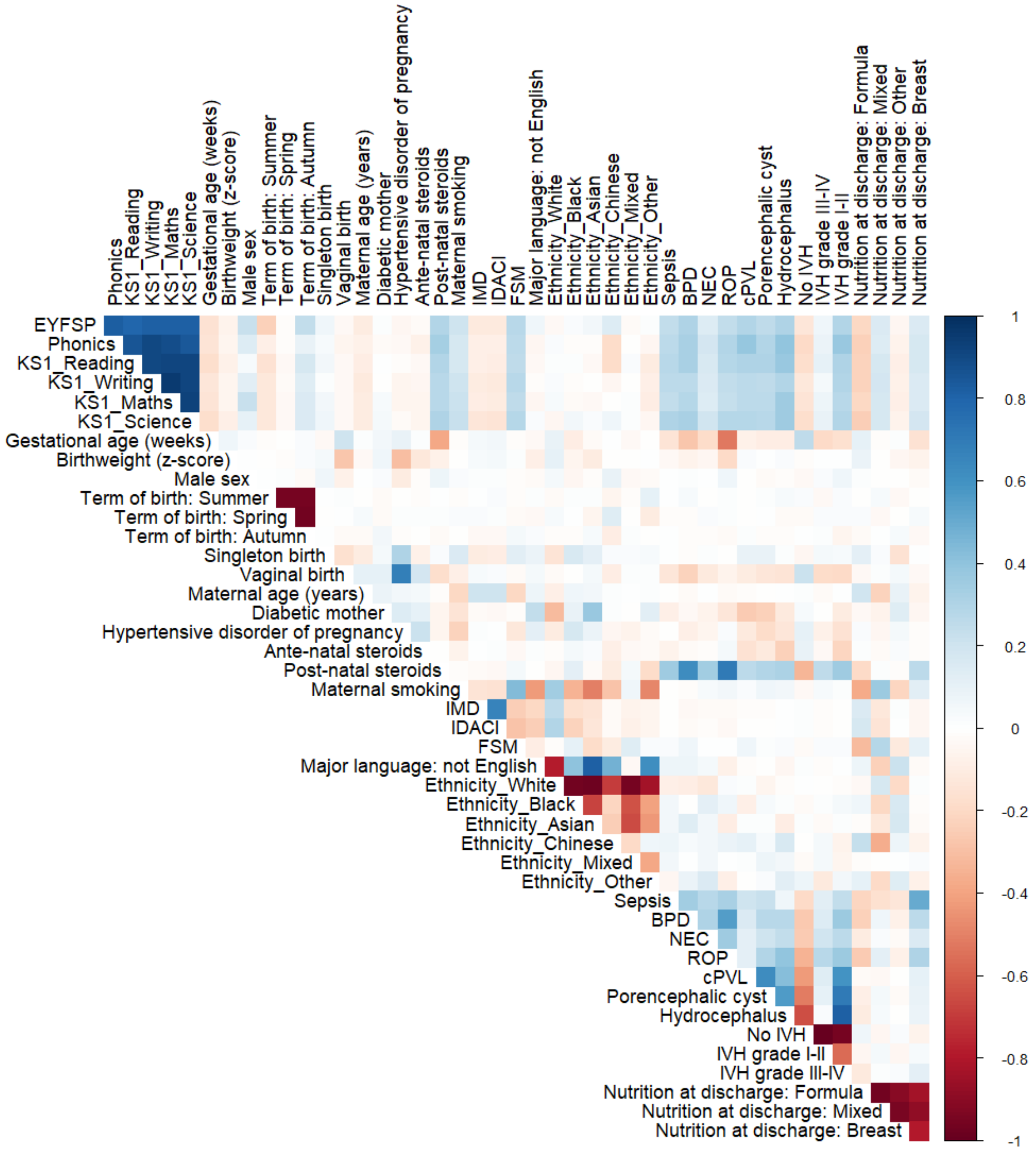

© 2026 Haider S et al. *JAMA Network Open*

eFigure 2. Participant Flow of Cohort Included in the Study Born Between 2008 or 2009 and 2011 or 2012 in England

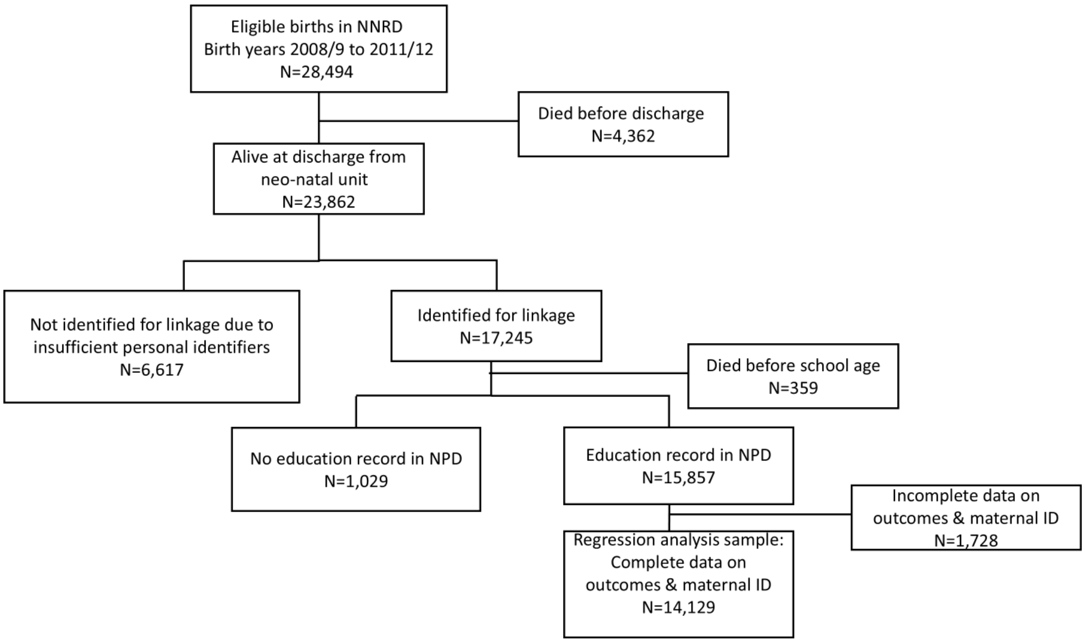

© 2026 Haider S et al. *JAMA Network Open*

eFigure 3. Distribution of Children Who Did Not Meet Expected Levels for Each Outcome in the Cohort and the Whole National Population

Comparison between educational outcome measures in the cohort and published national statistics for all children who were at school and undertook the assessments during the same time-period.

We derived the % who did not meet the expected level from annual figures published in reports by the Department for Education.[15,17]

The bars represent the mean and SD over four birth years (2008/09 to 2011/12). Figures show the percentage point difference between preterm cohort and national attainment.

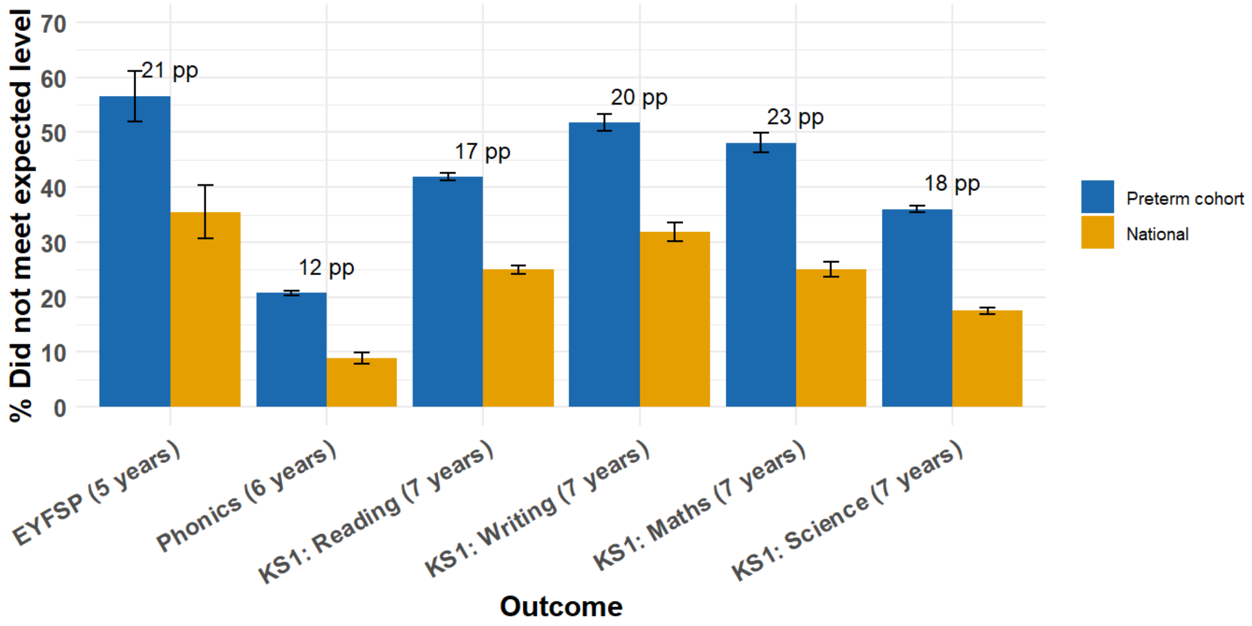

© 2026 Haider S et al. *JAMA Network Open*

eFigure 4. Proportion of Children Who Did Not Meet the Expected Level in Outcomes by Income Deprivation Affecting Children Index (IDACI) at Age 5 Years, Stratified by Gestational Age Category (23-26 Weeks and 27-31 Weeks) and the Overall Cohort

Trend tested using Cochran–Armitage test. P<0.001 for every line. IDACI 1=most deprived.

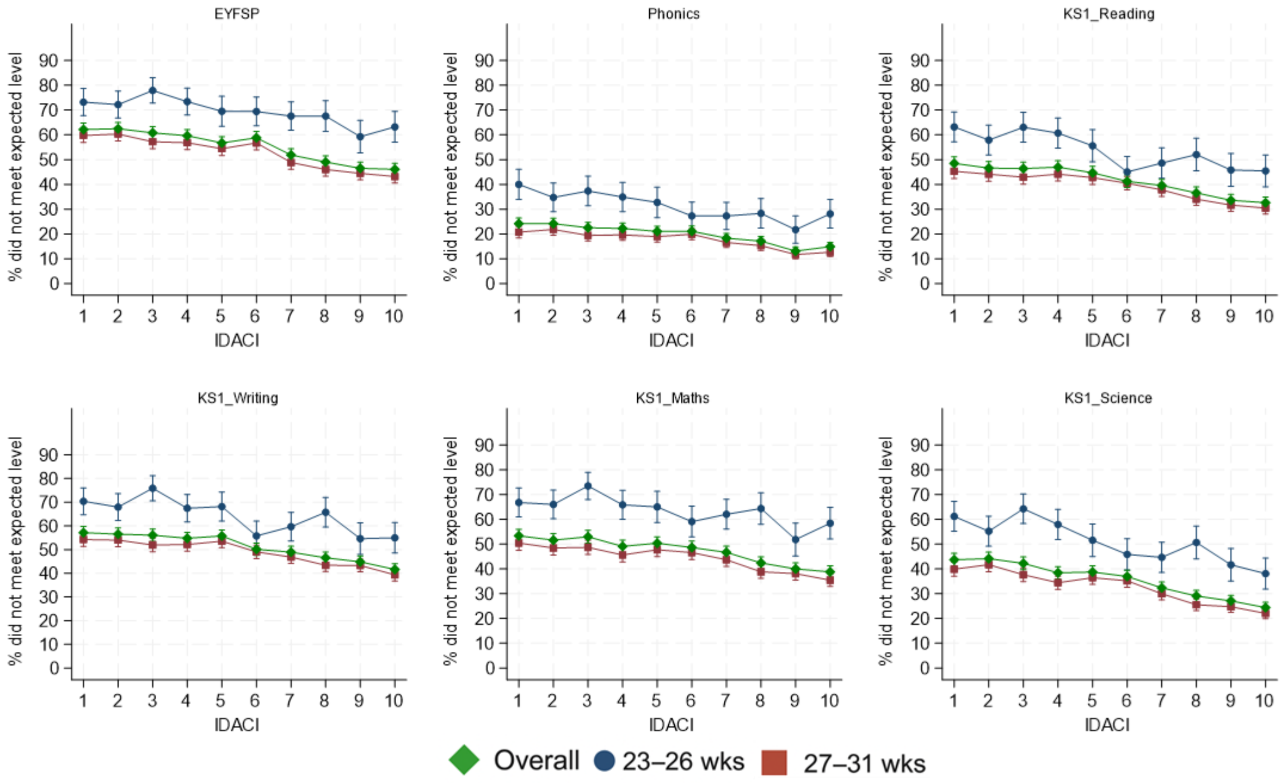

© 2026 Haider S et al. *JAMA Network Open*

eFigure 5. Proportion of Children Who Did Not Meet the Expected Level in Outcomes by Free School Meal Eligibility at Age 5 Years, Stratified by Gestational Age Category (23-26 Weeks and 27-31 Weeks) and the Overall Cohort

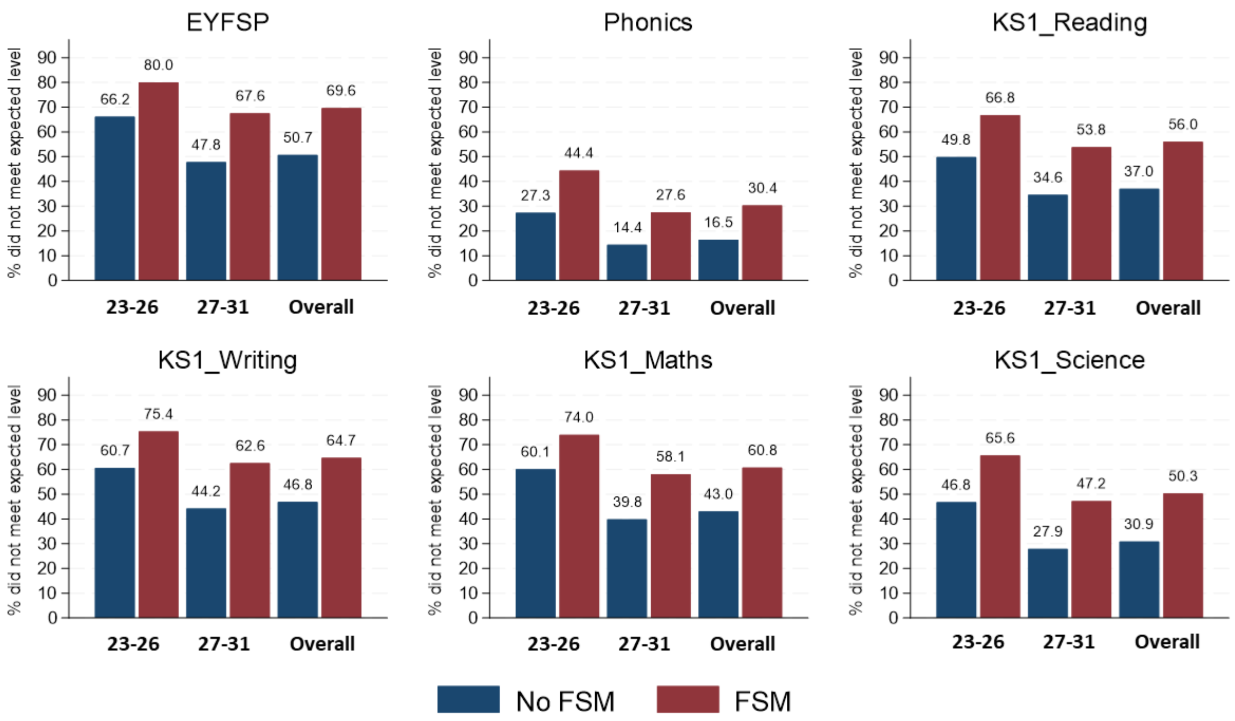

© 2026 Haider S et al. *JAMA Network Open*

eFigure 6. Forest Plot Showing the Unadjusted and Adjusted Associations Between Socioeconomic and Preterm Birth Factors With Educational Outcomes (Not Meeting the Expected Level in KS1 Writing or KS1 Science)

Odds ratio and 95% confidence intervals derived from GEE regression models.

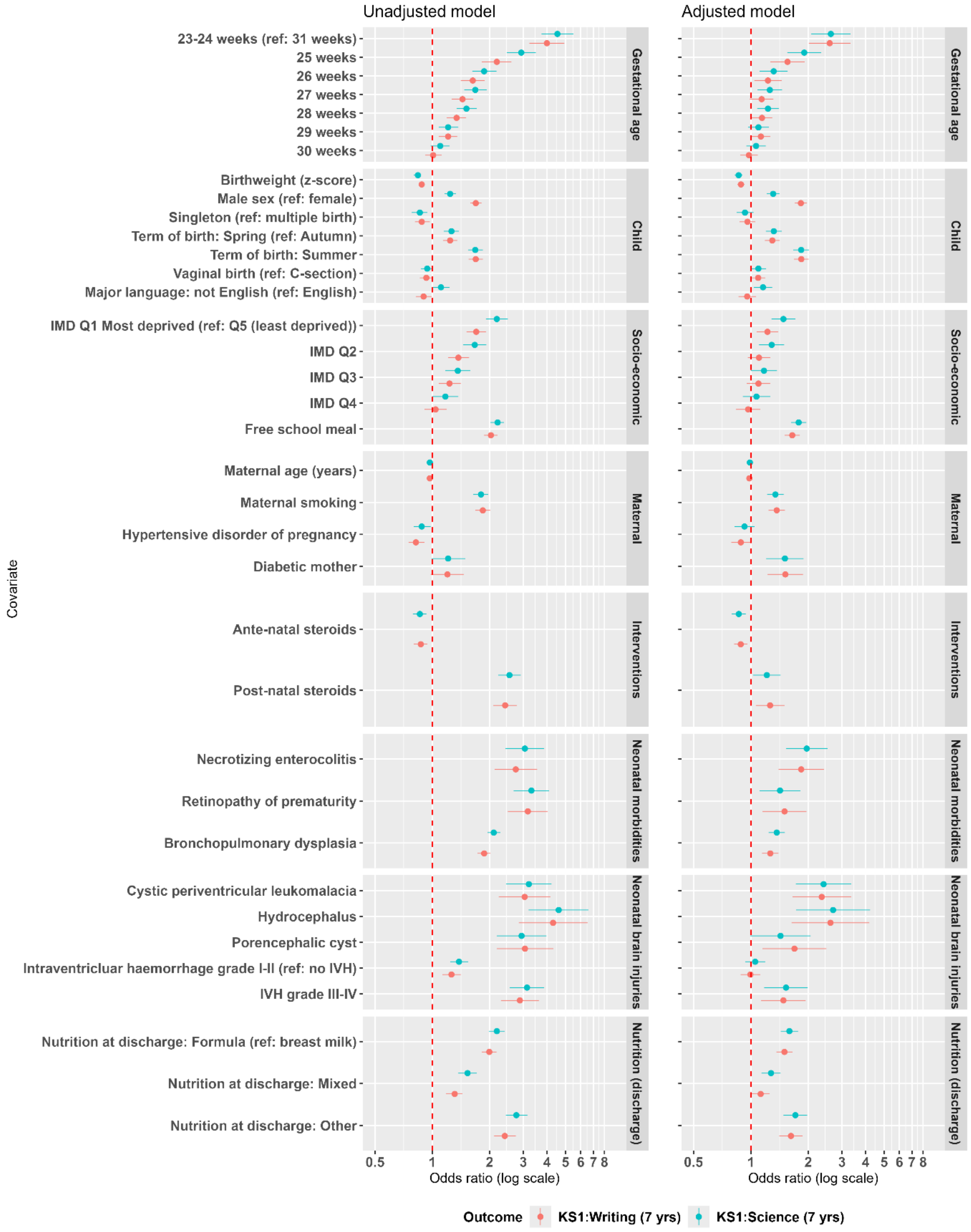

© 2026 Haider S et al. *JAMA Network Open*

eFigure 7. Estimated Probability of Not Meeting Expected Levels in Educational Outcomes Across Index of Multiple Deprivation (IMD) at Birth, Stratified by Gestational Age

Predicted probabilities derived from GEE interaction model including gestational age (GA), IMD, and their interaction. The interaction term was insignificant in each model at a p-value of 5%. The parallel lines indicate that GA and deprivation additively increased the probability of poor attainment. IMD 1= most deprived.

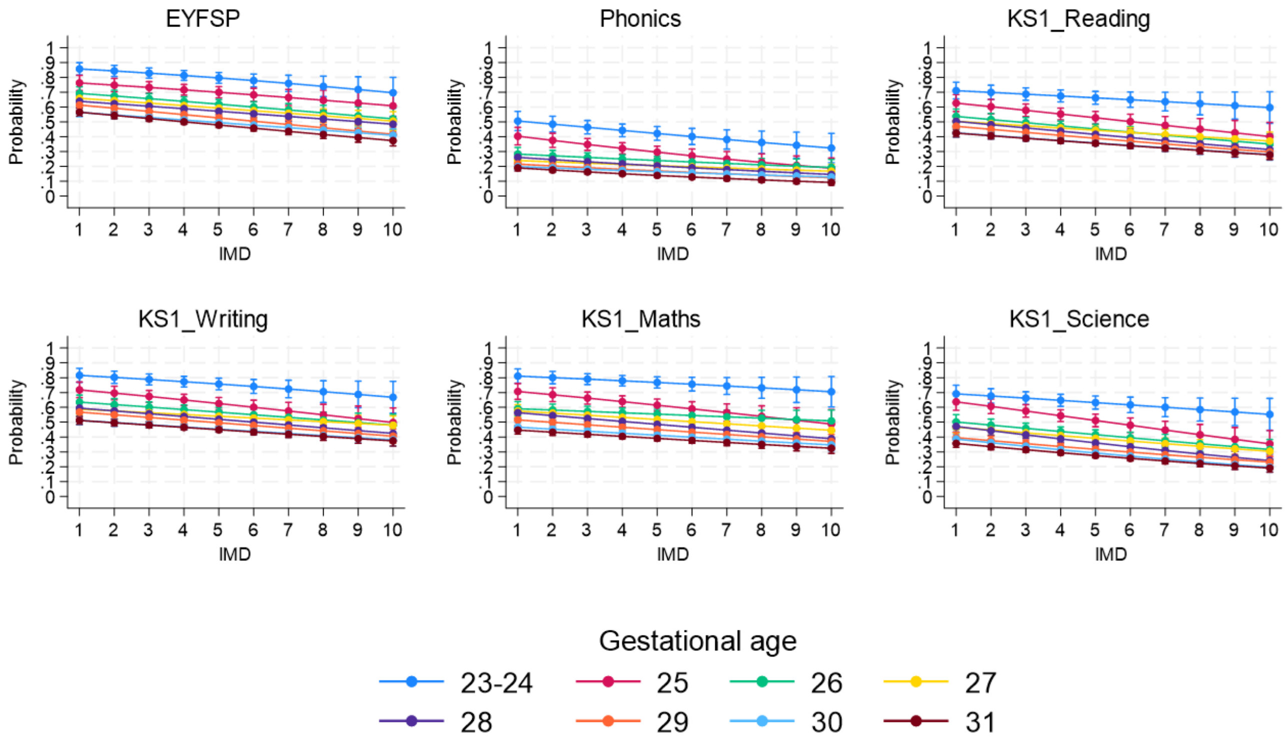

© 2026 Haider S et al. *JAMA Network Open*

eReferences

1. The Neonatal Data Analysis Unit. Vol. 2024 (Imperial College London).
2. Modi, N. Information technology infrastructure, quality improvement and research: the UK National Neonatal Research Database. *Transl Pediatr* **8**, 193-198 (2019).
3. Education, D.f. The National Pupil Database User Guide. (United Kingdom, 2015).
4. van Blankenstein, E., Aveline, A. & Battersby, C. A protocol for neoWONDER: Neonatal whole population data linkage to improve long-term health and wellbeing of preterm and sick babies. *PLoS One* **19**, e0305113 (2024).
5. RCPCH. National Neonatal Audit Programme: A guide to the 2023 audit measures. (London, 2023).
6. Conti-Ramsden, F., *et al.* The Contribution of Hypertensive Disorders of Pregnancy to Neonatal Unit Admissions and Iatrogenic Preterm Delivery at < 34(+0) Weeks' Gestation in the UK: A Population-Based Study Using the National Neonatal Research Database. *BJOG* **132**, 306-317 (2025).
7. Battersby, C., Longford, N., Mandalia, S., Costeloe, K. & Modi, N. Incidence and enteral feed antecedents of severe neonatal necrotising enterocolitis across neonatal networks in England, 2012-13: a whole-population surveillance study. *Lancet Gastroenterol Hepatol* **2**, 43-51 (2017).

© 2026 Haider S et al. *JAMA Network Open*
