# Supplementary material for: Health and Socioeconomic Factors in School Readiness and Achievement Among Children Born Very Preterm
Source: JAMA Netw Open. 2026 Jul 14;9(7):e2623068. doi: 10.1001/jamanetworkopen.2026.23068 (PMC13370314; doi:10.1001/jamanetworkopen.2026.23068)
Supplement: Supplement 2. — Data Sharing Statement [file jamanetwopen-e2623068-s002.pdf]

Data Sharing Statement

Data
Data available: No

Additional Information
Explanation for why data not available: The data underlying this study cannot be shared publicly due to data-sharing agreements with NHS Digital and the Department for Education in England.
